# Comparison of wild-type and high-risk PNPLA3 variants in a human biomimetic liver microphysiology system for metabolic dysfunction-associated steatotic liver disease precision therapy

**DOI:** 10.3389/fcell.2024.1423936

**Published:** 2024-09-11

**Authors:** Mengying Xia, Mahboubeh Varmazyad, Iris Pla-Palacín, Dillon C. Gavlock, Richard DeBiasio, Gregory LaRocca, Celeste Reese, Rodrigo M. Florentino, Lanuza A. P. Faccioli, Jacquelyn A. Brown, Lawrence A. Vernetti, Mark Schurdak, Andrew M. Stern, Albert Gough, Jaideep Behari, Alejandro Soto-Gutierrez, D. Lansing Taylor, Mark T. Miedel

**Affiliations:** ^1^ Drug Discovery Institute, University of Pittsburgh, Pittsburgh, PA, United States; ^2^ Department of Pathology, School of Medicine, University of Pittsburgh, Pittsburgh, PA, United States; ^3^ Center for Transcriptional Medicine, University of Pittsburgh, Pittsburgh, PA, United States; ^4^ Pittsburgh Liver Research Center, University of Pittsburgh, Pittsburgh, PA, United States; ^5^ Department of Computational and System Biology, School of Medicine, University of Pittsburgh, Pittsburgh, PA, United States; ^6^ Division of Gastroenterology, Hepatology and Nutrition, School of Medicine, University of Pittsburgh, Pittsburgh, PA, United States

**Keywords:** metabolic dysfunction-associated steatotic liver disease, microphysiology systems, patatin-like phospholipase domain-containing protein 3, precision medicine, reproducibility

## Abstract

Metabolic dysfunction-associated steatotic liver disease (MASLD) is a worldwide health epidemic with a global occurrence of approximately 30%. The pathogenesis of MASLD is a complex, multisystem disorder driven by multiple factors, including genetics, lifestyle, and the environment. Patient heterogeneity presents challenges in developing MASLD therapeutics, creating patient cohorts for clinical trials, and optimizing therapeutic strategies for specific patient cohorts. Implementing pre-clinical experimental models for drug development creates a significant challenge as simple *in vitro* systems and animal models do not fully recapitulate critical steps in the pathogenesis and the complexity of MASLD progression. To address this, we implemented a precision medicine strategy that couples the use of our liver acinus microphysiology system (LAMPS) constructed with patient-derived primary cells. We investigated the MASLD-associated genetic variant patatin-like phospholipase domain-containing protein 3 (PNPLA3) rs738409 (I148M variant) in primary hepatocytes as it is associated with MASLD progression. We constructed the LAMPS with genotyped wild-type and variant PNPLA3 hepatocytes, together with key non-parenchymal cells, and quantified the reproducibility of the model. We altered media components to mimic blood chemistries, including insulin, glucose, free fatty acids, and immune-activating molecules to reflect normal fasting (NF), early metabolic syndrome (EMS), and late metabolic syndrome (LMS) conditions. Finally, we investigated the response to treatment with resmetirom, an approved drug for metabolic syndrome-associated steatohepatitis (MASH), the progressive form of MASLD. This study, using primary cells, serves as a benchmark for studies using “patient biomimetic twins” constructed with patient induced pluripotent stem cell (iPSC)-derived liver cells using a panel of reproducible metrics. We observed increased steatosis, immune activation, stellate cell activation, and secretion of pro-fibrotic markers in the PNPLA3 GG variant compared to the wild-type CC LAMPS, consistent with the clinical characterization of this variant. We also observed greater resmetirom efficacy in the PNPLA3 wild-type CC LAMPS compared to the GG variant in multiple MASLD metrics, including steatosis, stellate cell activation, and the secretion of pro-fibrotic markers. In conclusion, our study demonstrates the capability of the LAMPS platform for the development of MASLD precision therapeutics, enrichment of patient cohorts for clinical trials, and optimization of therapeutic strategies for patient subgroups with different clinical traits and disease stages.

## Introduction

Metabolic dysfunction-associated steatotic liver disease (MASLD), previously named non-alcoholic fatty liver disease (NAFLD), is a chronic liver disease affecting ∼30% of the global adult population (Puri and Sanyal, 2012; Sass et al., 2005; Younossi et al., 2023; Battistella et al., 2024). The annual economic cost of the management of MASLD is projected at 100 billion dollars, affecting 30 million adults in the US, which is expected to increase to over 100 million by 2030 (Witkowski et al., 2022). MASLD is a complex, heterogeneous disease involving liver metabolic dysfunction associated with possible co-morbidities including T2D, obesity, and hypertension, with further heterogeneity from genetic risk factors (Eslam et al., 2020; Friedman et al., 2018; Loomba et al., 2021), as well as patient lifestyle and the environment. The progressive form of MASLD, metabolic dysfunction-associated steatohepatitis (MASH), is a major cause of hepatocellular carcinoma and is also the leading indication for liver transplantation in the United States (Paklar et al., 2023). The last decade has produced a major effort in developing MASLD/MASH therapeutics, with approximately 1,300 currently registered MASLD/MASH clinical trials (https://clinicaltrials.gov) (Chen, 2023; Fraile et al., 2021). The high failure rate in developing therapeutics for MASLD patients is due to a combination of factors including a) the complex, heterogeneous nature of the disease, including a variety of possible co-morbidities; b) genotypic differences in key MASLD-associated genes; c) patient lifestyle and environment; and d) the historical reliance on animal models of disease that do not fully recapitulate the human disease complexity and heterogeneity (Flessa et al., 2022; Hebbard and George, 2011; Jahn et al., 2019; Takahashi et al., 2012; Van Herck et al., 2017; Zhong et al., 2024). Recently, resmetirom (Rezdiffra™) became the first approved drug for MASH with stage 2 or 3 fibrosis; however, it only impacts approximately 25% of the patients treated in clinical trials (Harrison et al., 2024).

Genetic factors contribute to MASLD development and progression (Romeo et al., 2020; Eslam et al., 2018). A correlation between increased risk factors and MASLD patients carrying specific gene variants, including patatin-like phospholipase domain-containing protein 3 (PNPLA3) rs738409, MBOAT7 rs641738, TM6SF2 rs58542926, and GCKR rs780094, is well known (Sulaiman et al., 2022; Mahmoudi et al., 2024). Among these noted genetic variants, evidence shows that the human PNPLA3 gene rs738409 C>G polymorphism (PNPLA3 rs738409/I148M) has a strong correlation to increased MASLD severity, including hepatic steatosis, fibrosis, cirrhosis, and hepatocellular carcinoma (Romeo et al., 2008; Sookoian and Pirola, 2011; Sookoian et al., 2009; Valenti et al., 2010; Liu et al., 2014; Dong, 2019; Dongiovanni et al., 2013). *In vitro* experiments show that PNPLA3 exhibits triacylglycerol hydrolase, acyltransferase, and transacylase activities, which regulate lipid droplet remodeling in both hepatocytes and hepatic stellate cells (Pingitore et al., 2014; Kumari et al., 2012; Pirazzi et al., 2014). The PNPLA3 C>G (GG) variant results in a reduction in fatty acid hydrolysis and impaired mobilization of triglycerides, resulting in hepatic triglyceride accumulation (Romeo et al., 2008; Pingitore and Romeo, 2019). Although largely unknown, it has been observed that patients carrying the GG variant show a differential response to some drug treatments compared to patients carrying wild-type (CC) PNPLA3, including omega-3 fatty acid docosahexaenoic acid (DHA), statins, and dipeptidyl peptidase-4 (DPP-4) inhibitors (Nobili et al., 2013; Scorletti et al., 2015; Wang et al., 2018; Hendriks et al., 2023). Thus, this differential response provides an initial basis for stratifying MASLD patients according to their PNPLA3 genotype, offering an initial strategy for implementing a precision medicine-based approach for investigating disease progression and assessing drug responses within specific PNPLA3 cohorts.

Animal models to study MASLD progression and evaluate key disease-associated genetic variants such as the PNPLA3 GG polymorphism have been characterized with various metrics demonstrating increased steatosis, inflammation, fibrogenesis, oxidative stress, and insulin resistance yet do not fully recapitulate the complex and heterogeneous progression of the human disease (Flessa et al., 2022; Hebbard and George, 2011; Jahn et al., 2019; Takahashi et al., 2012; Van Herck et al., 2017; Zhong et al., 2024). Human microphysiology systems (MPSs) are experimental models designed to recapitulate the structure and both normal- and disease-state physiology of tissues and organs to serve as a complement to existing animal models (Hargrove-Grimes et al., 2021; Hargrove-Grimes et al., 2022). MPSs are 3D microfluidic platforms composed of multiple cell types that mimic the overall organ structure and provide cell-to-cell communication using human primary cells, immortalized cell lines, and induced pluripotent stem cells (iPSCs) (Taylor et al., 2019; Gough et al., 2021; Low et al., 2021). Recently, multiple human liver MPSs have evolved and been implemented to study the mechanisms of MASLD pathogenesis and serve as drug-testing platforms (Gough et al., 2021; Feaver et al., 2016; Kostrzewski et al., 2017; Kostrzewski et al., 2020; Kostrzewski et al., 2021; Lefever et al., 2022; Saydmohammed et al., 2021; Li et al., 2018; Yang et al., 2023; Slaughter et al., 2021; Strobel et al., 2021; Kermanizadeh et al., 2022; Wang et al., 2020; Telles-Silva et al., 2022; Qi et al., 2022; Qi et al., 2023; Ouchi et al., 2019; Liu et al., 2023; Yuan et al., 2023; Kukla and Khetani, 2021). We implemented the liver acinus microphysiology system (LAMPS), a structured biomimetic, that is a 3D-layered model constructed through a combination of sequential cell layering and cell-to-cell self-organization using a hybrid model of four key liver cell types, namely, primary hepatocytes, liver sinusoidal endothelial cells (LSECs), and two well-established cell lines for hepatic stellate cells (LX-2) and Kupffer-like cells (THP-1), and maintained under flow to mimic either zone-1 or zone-3 oxygen tensions (Lee-Montiel et al., 2017; Miedel et al., 2019; Vernetti et al., 2016) (Supplementary Figure S1). The LAMPS has been tested and reproduced by the Texas A&M Tissue Chip Validation Center (Tex-Val), one of the National Center for Advancing Translational Sciences (NCATS)-funded Tissue Chip Testing Centers (TCTC), and has demonstrated reproducible features for hepatic function (Lefever et al., 2022; Saydmohammed et al., 2021; Vernetti et al., 2016; Vernetti et al., 2017; Beaudoin et al., 2023; Sakolish et al., 2021a; Sakolish et al., 2021b; Gough et al., 2016; Schurdak et al., 2020).

**FIGURE 1:**
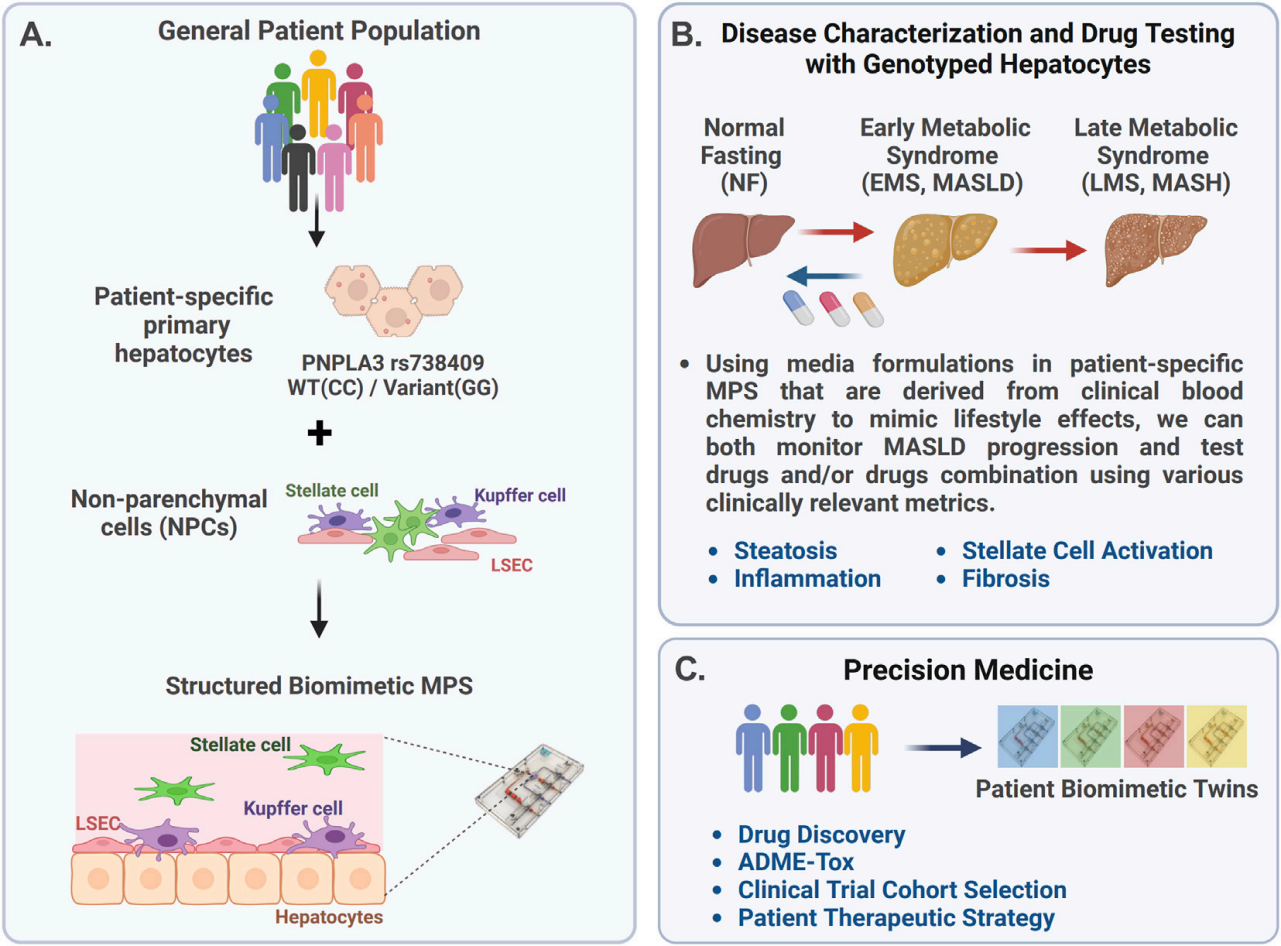
Overview of the precision medicine approach used to investigate genotype-specific MASLD progression and drug response using PNPLA3 genotyped patient-derived cells in the liver acinus microphysiology system (LAMPS). **(A)** MASLD progression is complex due to multiple factors, including genetics, environment, and lifestyle, resulting in patient heterogeneity. The PNPLA3 rs738409 GG variant is highly associated with MASLD susceptibility (Romeo et al., 2008; Sookoian and Pirola, 2011; Rotman et al., 2010; Xu et al., 2015). To study this high-risk variant, we started with the genotyped PNPLA3 rs738409 GG variant and CC wild-type primary hepatocytes to use as a benchmark for studies using iPSC-derived liver cells (de I’Hortet et al., 2019; Takeishi et al., 2020). These genotyped primary hepatocytes were cultured with nonparenchymal cells (NPCs) including liver sinusoidal endothelial cells (LSECs), stellate cells, and Kupffer cells in the LAMPS (Lefever et al., 2022; Saydmohammed et al., 2021; Vernetti et al., 2016). **(B)** MASLD progression in the LAMPS was driven with specific medium formulations to mimic lifestyle effects, as previously described (Saydmohammed et al., 2021), and monitored through various clinically relevant metrics, including steatosis, stellate cell activation, inflammation, and fibrosis. Additionally, the efficacy of recently FDA-approved resmetirom (Rezdiffra™) (Harrison et al., 2024; Harrison et al., 2019) was assessed in the LAMPS, focusing on the early stage of MASLD progression using the EMS medium. **(C)** Results from these studies can then inform a precision medicine strategy that implements the use of patient-derived iPSCs to generate patient biomimetic twins (PBTs) for drug testing and development, as well as clinical trial patient cohort selection (Gough et al., 2021).

The complex and heterogeneous nature of MASLD progression suggests that a precision medicine approach is required for improved success in developing therapeutics, segmenting MASLD patients into specific cohorts for enrollment in clinical trials, and optimizing therapeutic strategies for specific patient cohorts. Thus, a critical question for clinicians is how to better predict responders more accurately from non-responders for any given MASLD therapeutic. The use of an MPS in precision medicine is rapidly evolving, with advancements in the fabrication, materials, and use of patient-derived cells, including various stem cell technologies. By incorporating patient-derived cells into the MPS, it is possible to generate precision models of complex diseases, allowing for the testing of different drugs or treatment combinations directly on the patient cells to identify effective therapies. However, implementing the use of an MPS into precision medicine platforms is an iterative process as a critical challenge is to validate the functionality and reproducibility of patient-specific models produced with patient-derived iPSCs. Thus, our strategy (Figure 1) first involves constructing MASLD LAMPS models with a hybrid cell configuration using genotyped primary hepatocytes, primary LSECs, and well-established cell lines for Kupffer and stellate cells (Figure 1A). Using media formulations derived from clinical blood chemistry indicators in the LAMPS, we monitored both MASLD progression and test drug efficacy using a panel of disease-relevant metrics (Figure 1B) (Lefever et al., 2022; Saydmohammed et al., 2021). These initial studies using the hybrid LAMPS model serve as a critical benchmark for future MASLD progression and drug-testing studies then performed in the LAMPS constructed with patient-derived iPSCs, which are referred to as “patient biomimetic twins” (PBTs) (Gough et al., 2021). The results from these studies coupled with the use of patient information and clinomics (e.g., proteomics, transcriptomics, and metabolomics) inform a precision medicine strategy using PBTs for drug testing and development to inform clinical trial cohort selection (Figure 1C) (Gough et al., 2016).

In this study, we aimed to determine whether the MASLD LAMPS constructed with the genotyped PNPLA3 GG variant or CC wild-type primary hepatocytes exhibited differences in MASLD progression and drug efficacy. MASLD disease phenotypes in the LAMPS were driven using medium formulations to mimic disease progression from normal fasting (NF) to early metabolic syndrome (EMS) and then late metabolic syndrome (LMS) for 8 days (Lefever et al., 2022; Saydmohammed et al., 2021; Gough et al., 2016). MASLD progression and response to resmetirom treatment were monitored through multiple phenotypic metrics, including steatosis, pro-inflammatory cytokine production, stellate cell activation, and fibrosis.

## Materials and methods

**Cell sources and initial culture.** Cryopreserved primary human hepatocytes were genotyped for PNPLA3 rs738409(G) using TaqMan^®^ SNP Genotyping Assays (Life Technologies, Assay ID C_7241_10, 4351379) according to the manufacturer’s protocol (Supplementary Figure S2). Genotyping was performed by extracting genomic DNA using the DNeasy Blood & Tissue Kit (QIAGEN, Hilden, Germany), following the manufacturer’s instructions. Genomic DNA samples were genotyped using TaqMan SNP Genotyping Assays for PNPLA3 rs738409 (Thermo Fisher Scientific, Waltham, MA). Amplification and genotype clustering were performed using a StepOnePlus System (Applied Biosystems, Foster City, CA). PNPLA3 GG homozygotes and CC homozygotes with >90% viability, post-thaw re-plating efficiency >90%, and suitable for long-term culture were selected and purchased from Discovery Life Sciences (Gentest^®^ 999Elite^™^ human hepatocytes; catalog #82006); GG variant lots: HH1142 and HH1072; CC wild-type lots: HH1136 and HH1178 (Thermo Fisher Scientific) (Human Plateable Hepatocytes, Hu8391 (CC, wild-type), catalog # HMCPTS). Isolated primary human LSECs were purchased from LifeNet Health (NPC-AD-LEC-P1). The human monoblast cell line, THP-1, used to generate Kupffer-like cells, was purchased from ATCC (TIB-202). Prior to seeding into the LAMPS, THP-1 cells were treated with phorbol 12-myristate 13-acetate (PMA; Sigma-Aldrich, 524400), which induces THP-1 cells to differentiate into macrophage-like cells and causes inhibition of cell growth (Traore et al., 2005). LX-2 human stellate cells were purchased from Sigma-Aldrich (SCC064). LSECs were cultured in endothelial cell basal medium-2 (EBM-2, Lonza, CC-3162). THP-1 cells were cultured in suspension in RPMI 1640 Medium (Cytiva, SH30096.FS) supplemented with 10% regular fetal bovine serum (FBS; Corning, MT35010CV), 100 μg/mL penicillin–streptomycin (Cytiva, SV30010), and 2 mM L-glutamine (Cytiva, SH30034.01). LX-2 cells were cultured in Dulbecco’s modified Eagle’s medium (DMEM; Thermo Fisher Scientific, 11965118) supplemented with 2% FBS, 100 units/mL penicillin, and 100 μg/mL streptomycin.

**LAMPS assembly and maintenance**. LAMPS studies were carried out as previously described for model assembly (Gough et al., 2021; Lefever et al., 2022; Saydmohammed et al., 2021; Lee-Montiel et al., 2017; Miedel et al., 2019; Vernetti et al., 2016) (Supplementary Figure S1). A more detailed description of the LAMPS assembly is given in Supplementary Methods. In brief, LAMPS models were constructed using the HAR-V single-channel device (SCC-001) from Nortis, which is composed of four key liver cell types and used at the following cell densities: primary cryopreserved human hepatocytes (2.75 × 10^6^ cells/mL), primary LSECs (1.5 × 10^6^ cells/mL), THP-1 (0.4 × 10^6^ cells/mL), and LX-2 (0.2 × 10^6^ cells/mL). The percentages of hepatocytes (56%), THP-1 (18%), LSECs (22%), and LX-2 (4%) cells are consistent with the scaling used in our previously published models (Lefever et al., 2022; Lee-Montiel et al., 2017; Miedel et al., 2019; Vernetti et al., 2016). The interior of the devices was coated with 100 μg/mL bovine fibronectin (Sigma-Aldrich, F1141) and 150 μg/mL rat-tail collagen (Corning, 354249) in PBS prior to cell seeding. For all steps involving the injection of media and/or cell suspensions into LAMPS devices, 100–150 μL per device was used to ensure complete filling of fluidic pathways, chamber, and bubble traps. The devices were then overlaid with 2.5 mg/mL rat-tail collagen I (Corning) and maintained with the perfusion of different conditions for 8 days at a flow rate of 5 μL/h to recapitulate zone-3 oxygen tension (Lee-Montiel et al., 2017).

**Formulation of normal fasting, early metabolic syndrome, and late metabolic syndrome media**. The media were formulated as previously described (Saydmohammed et al., 2021) to create disease progression from NF to EMS or MASLD and LMS or late-stage MASLD. The media were formulated with glucose-free William’s E Medium (Thermo Fisher Scientific, ME18082L1) as the base medium supplemented with physiologically relevant levels of glucose (Sigma-Aldrich, G8644), insulin (Thermo Fisher Scientific, 12585014), glucagon (Sigma-Aldrich, G2044), oleic acid (Cayman Chemical, 29557), palmitate acid (Cayman Chemical, 29558), and molecular drivers of disease, including TGF-β1 (Thermo Fisher Scientific, PHG9214) and lipopolysaccharide (Sigma-Aldrich, L2654), as shown in Supplementary Table S1.

**96-well plate LAMPS assembly and maintenance**. The 96-well plate LAMPS was used for the lipid peroxidation analysis. The 96-well plate LAMPS was constructed as previously described (Miedel et al., 2019). In brief, the 96-well plate LAMPS followed the same percentage of each cell type in the LAMPS model. Hepatocytes (50,000 cells/well) were plated in a collagen I-coated clear-bottom 96-well plate (Thermo Fisher Scientific, 08-774-307). A porcine liver extracellular matrix (LECM) (400 μg/mL) was added on the top of the hepatocytes to create a thin matrix layer. A mixture of LSECs (0.54 × 10^6^ cells/mL) and THP-1 (0.28 × 10^6^ cells/mL) was added on the top of the LECM; finally, an overlay of LX-2 (0.1 × 10^6^ cells/mL) suspended in a 2.5 mg/mL solution of rat-tail collagen (pH 7.2) was added. The 96-well plate LAMPS was maintained in 100 μL LAMPS perfusion media. The media were replaced every 48 h during the experimental time course.

**Image and analysis of the lipid peroxidation (LPO) level**. The BODIPY 581/591 C11 (lipid peroxidation sensor, Thermo Fisher Scientific, D3861) was added to the 96-well co-culture model on day 5 at a final concentration of 5 µM in the appropriate medium (NF, EMS, or LMS) and incubated for 30 min at 37°C. Hydrogen peroxide was used as a positive control to induce LPO. Prior to imaging, the cells were washed twice with PBS and then returned to their original culture medium. The signals from both oxidized C11 (488 nm/FITC, laser/filter) and non-oxidized C11 (561 nm/Cy3, laser/filter) were monitored. Maximum projection images were generated. The ratio of the mean fluorescence intensity (MFI) of FITC to MFI of Cy3 was calculated as the relative LPO level by using Harmony software (Revvity, v5.1).

**Drug treatment**. Stock solutions (50 mM) of resmetirom (MGL-3196, MedChemExpress) were prepared from powder by resuspending in DMSO (Sigma-Aldrich, 34869). The stock solution was first diluted in DMSO to obtain a 5 mM solution and then further diluted in perfusion media (EMS) to obtain a final concentration of 1 μM for drug treatment and 0.02% DMSO for the vehicle control. The LAMPS was then maintained in the EMS medium containing either the vehicle control or the 1 μM resmetirom for 8 days at a flow rate of 5 μL/h. The images and collected efflux were analyzed, and the drug-treated devices were normalized to their respective vehicle control (described below). The drug-binding capability of the polydimethylsiloxane (PDMS)-containing LAMPS device was assessed as previously described (Lefever et al., 2022; Miedel et al., 2019; Vernetti et al., 2016) using perfusion flow tests and mass spectrometry analysis of the efflux collected from cell-free LAMPS devices after 72 h of flow to determine the overall effective concentration of each compound compared to the starting concentration of the drug in the perfusion medium.

**LipidTOX labeling and αSMA immunofluorescence**. Cells were fixed with 4% paraformaldehyde (Thermo Fisher Scientific, AA433689M) in PBS for 30 min and then washed twice with PBS for 10 min at room temperature. Following fixation, LipidTOX Deep Red Neutral Lipid Stain (1:500; Invitrogen, H34477) and mouse monoclonal anti-α-smooth muscle actin (αSMA) antibody (1:100; Sigma-Aldrich, A2547) in PBS were perfused into devices and incubated overnight at 4°C. The following day, the devices were washed twice with PBS and then incubated for 2 h with the Alexa Fluor 488 goat anti-mouse (1:250; Invitrogen, A-11029) secondary antibody and Hoechst (5 μg/mL, Invitrogen, H1399) at room temperature. Lastly, the devices were washed three times with PBS before imaging. If not imaged immediately, the samples were stored at 4°C and imaged within 1 week.

**Confocal imaging and analysis**. Confocal imaging was performed using the Phenix High-Content Imaging platform (Revvity), using a 40×/0.75 hN A air objective. z-stacks of 70-μm distance (3 μm spacing between slices) were obtained across an array of 3 × 7 adjacent fields covering an area of 2.15 mm^2^ in the LAMPS device. Images for each condition were acquired using the same exposure time and laser power settings to ensure that intensity values were ∼50–90% of the total dynamic range. Image analysis was performed using custom analysis protocols developed in the Harmony (Revvity, v5.1) software package.

**Measurement of steatosis**. Steatosis was measured after the completion of the experimental time course on day 8. The nuclei were visualized by staining with Hoechst and acquired using a 405-nm laser and DAPI filter. The LipidTOX signal was acquired using a 640-nm laser and Cy5 filter. Imaging parameters (exposure time and laser power) were set using the EMS because this condition demonstrated the most LipidTOX labeling. The LipidTOX fluorescence intensity volume was calculated using the 3D analysis method in Harmony software (Revvity, v5.1). Lipid droplet objects were identified using a local thresholding method and region scaling parameter defined by Harmony software. This method creates a region or set of regions covering all pixels of the image with an intensity higher than their locally surrounding intensity.

**Measurement of stellate cell activation**. Immunofluorescence for LX-2 cell expression of αSMA was performed after the completion of the experimental time course on day 8. The nucleus signal was acquired using the 405-nm laser and DAPI filter. The αSMA signal was acquired using a 488-nm laser and FITC filter. The image analysis of LX-2 αSMA expression was quantified using a maximum intensity projection in Harmony software (Revvity, v5.1). The particle detection function was then applied with a size exclusion setting of 100 μm^2^ to exclude non-specific labeling. The MFI of FITC was calculated as the integrated intensity of the αSMA signal.

**Efflux collection and biochemical measurements**. Albumin (ALB), urea (BUN), lactate dehydrogenase (LDH), and pro-collagen I alpha 1 (COL1A1) levels were measured, as previously described (Lefever et al., 2022; Saydmohammed et al., 2021; Miedel et al., 2019; Vernetti et al., 2016). In brief, efflux from the LAMPS was collected on days 2, 4, 6, and 8. ALB assays were performed in 1:100 efflux dilutions using enzyme-linked immunosorbent assay (ELISA) using commercial antibodies (Bethyl Laboratories, A80-129A and A80-129P) and an ELISA accessory kit (Bethyl Laboratories, E101) with a human albumin standard prepared in-house (MilliporeSigma, 126658). The COL1A1 level was measured using the human pro-collagen 1A1 ELISA kit (R&D Systems, cat. no. DY6220-05) in a 1:50 efflux dilution. The BUN level was measured using the Stanbio BUN Liquid Reagent for Diagnostic Set (Stanbio Laboratory, cat. no. SB-0580-250). The LDH level was measured using the CytoTox 96 Non-Radioactive Cytotoxicity Assay (Promega, cat. no. G1780). The protocols for the BUN and LDH assay were modified to a 384-well microplate format with no efflux dilution.

**Multiplex immunoassays**. The levels of IL-6, IL-8, and MCP-1 were determined in efflux collected on day 8 using a custom version of the Human XL Cytokine Performance Panel (R&D Systems). Sex hormone-binding globulin (SHBG) levels were quantified using the Human XL Cytokine Discovery Panel (R&D Systems). Assays were completed according to the manufacturer’s instructions at the University of Pittsburgh Cancer Proteomics Facility Luminex^®^ Core Laboratory on the xMAP platform. All the cytokine target profiling experiments were performed from efflux obtained from n = 3 devices for each experimental condition.

**Statistical analysis**. Inter-study reproducibility analysis was performed in Eve Analytics™ (previously, BioSystics Analytics Platform and the MPS Database) using the Pittsburgh Reproducibility Protocol (PReP) (Schurdak et al., 2020; Miedel et al., 2024). One-way ANOVA was used to compare three or more studies run under identical conditions using the same cell lot. A pooled *t*-test was used when only duplicate identical studies were run. The reproducibility status was determined based on the *p*-value with *p*-values ≥ 0.1 showing excellent reproducibility, 0.05–0.1 considered acceptable reproducibility, and <0.05 being poorly reproducible.

Data analyzing genotype-specific differences across medium conditions and drug treatments were analyzed and plotted in R (version 4.3.1). The data were obtained with a minimum of n = 3 LAMPSs for each patient cell lot for each of the three medium conditions and drug treatments. The data are plotted as the mean ± standard error of mean (SEM). Statistical significance was assessed by ANOVA with Tukey’s test for multiple comparisons with *p* < 0.05 considered statistically significant.

## Results

### Increased steatosis is observed in the PNPLA3 rs738409 GG variant LAMPS compared to the CC wild-type LAMPS consistent with the clinical characterization of this polymorphism

To initiate the precision medicine approach outlined in Figure 1, we used specific lots of patient hepatocytes that were genotyped to identify either the PNPLA3 CC wild-type or rs738409 GG PNPLA3 high-risk variant (Supplementary Figure S2; Supplementary Table S2). These genotyped primary hepatocytes were used in combination with primary LSECs and cell lines for hepatic stellate cells (LX-2 cell line) and Kupffer-like cells (THP-1 cell line) to construct the LAMPS (Supplementary Figure S1A). We examined the specific impact of the PNPLA3 rs738409 GG variant on steatosis, cytokine secretion, stellate cell activation, and fibrosis over an 8-day time course using previously published media formulated to recapitulate MASLD phenotypes (Supplementary Table S1; Supplementary Figure S1B) (Lefever et al., 2022; Saydmohammed et al., 2021).

To evaluate genotype-specific differences in both model functionality and cytotoxicity under each medium condition, the PNPLA3 GG variant and CC wild-type LAMPS were maintained in either NF, EMS, or LMS medium for 8 days. Over the experimental time course, similar nucleus counts were observed on day 8 between the PNPLA3 GG variant and CC wild-type LAMPS under each medium condition (Supplementary Figure S3).

Medium efflux samples were collected on days 2, 4, 6, and 8 to measure the secretion of ALB and BUN for LAMPS functionality and LDH for LAMPS cytotoxicity (Supplementary Figure S4). Although ALB secretion was higher in the EMS medium than that in NF and LMS media, consistent with our previous work (Lefever et al., 2022; Saydmohammed et al., 2021), no significant differences in ALB secretion were observed between the PNPLA3 GG variant and CC wild-type LAMPS under any medium condition (Supplementary Figure S4A), demonstrating similar overall model functionality. However, a significant increase in BUN secretion was observed on days 4, 6, and 8 in the EMS medium in the PNPLA3 GG LAMPS compared to the CC wild-type LAMPS, suggesting an overall increase in protein catabolism in the LAMPS constructed with the high-risk variant under this medium condition (Supplementary Figure S4B). In addition, a significant increase in LDH secretion was observed in both NF and EMS media on day 8 and on days 4 and 6 in the LMS medium, suggesting an overall increase in cytotoxicity in the PNPLA3 GG LAMPS, consistent with its characterization as a high-risk variant associated with an increased risk for MASLD progression and liver damage (Supplementary Figure S4C) (Mann and Anstee, 2017; Mann et al., 2020; Min et al., 2014).

**FIGURE 2:**
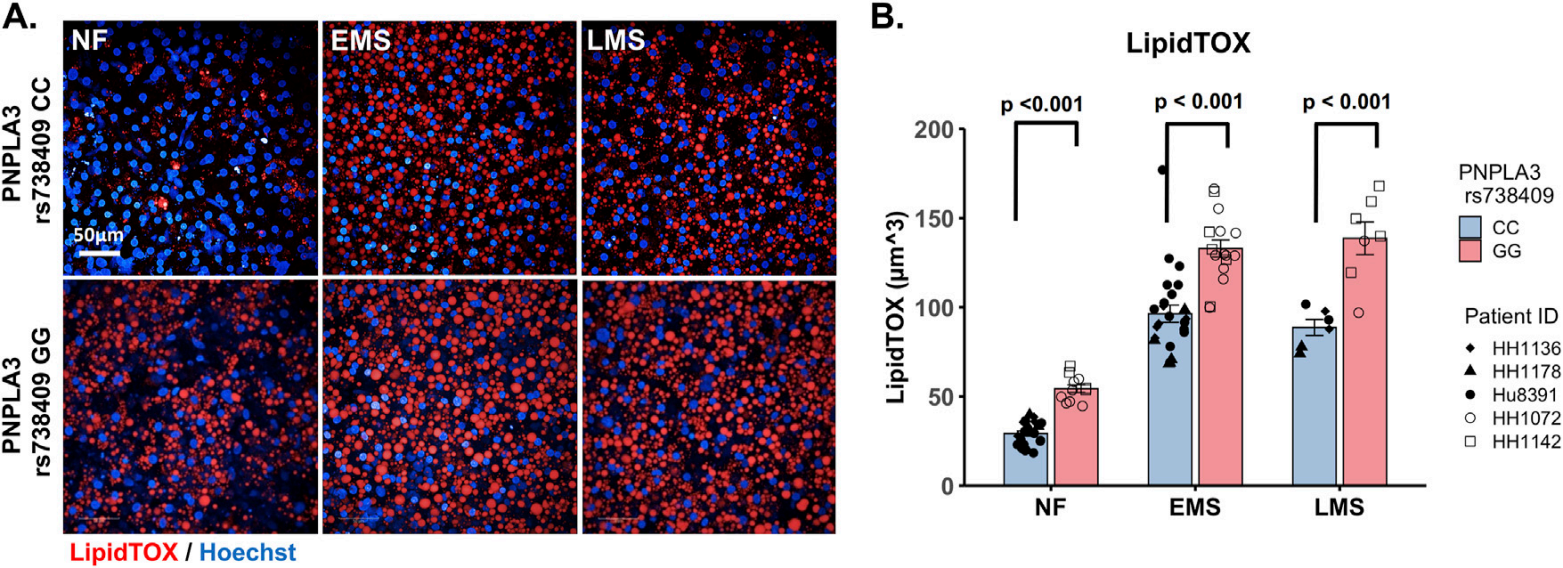
Increased steatosis in the PNPLA3 rs738409 GG variant LAMPS compared to the CC wild-type LAMPS is consistent with the clinical characterization of the GG variant. **(A)** Representative images of the LipidTOX-labeled PNPLA3 LAMPS maintained in NF, EMS, and LMS media. Magnification: 40×; scale is 50 µm. **(B)** Steatosis was quantified by quantitative fluorescence imaging of LipidTOX-labeled samples under each medium condition. Significant increases in steatosis were observed in the PNPLA3 GG variant LAMPS compared to the CC wild-type LAMPS under all three medium conditions, consistent with the clinical characterization of this high-risk variant (Romeo et al., 2008). Data were obtained on day 8 with a minimum of n = 3 LAMPSs from each patient lot for each condition and plotted as the mean ± SEM. Statistical significance was assessed by ANOVA with Tukey’s test. *p*-value <0.05 was considered statistically significant. Statistical comparisons across medium conditions within each genotype were also performed, as shown in Supplementary Figure S5.

As excess hepatic fat content is strongly associated with the PNPLA3 GG variant (Romeo et al., 2008; Mann and Anstee, 2017), we next evaluated genotype-specific effects on hepatocellular steatosis using quantitative fluorescence imaging to quantify LipidTOX staining in the PNPLA3 GG variant and CC wild-type LAMPS that were maintained for 8 days in either NF, EMS, or LMS media (Figure 2; Supplementary Figure S5). Compared to the CC wild-type LAMPS, significant increases in steatosis were observed in the PNPLA3 GG variant LAMPS under all three medium conditions, indicating that increased hepatic lipid content is associated with the GG variant under both the baseline NF condition and early and late-stage MASLD (EMS and LMS) medium conditions (Figures 2A,B), consistent with the clinical characterization that this high-risk variant is associated with increased susceptibility to hepatic steatosis (Romeo et al., 2008; Mann et al., 2020). In addition, significant increases in steatosis were observed under both the EMS and LMS medium conditions compared to the NF medium for both the PNPLA3 CC wild-type and GG LAMPS (Supplementary Figure S5), demonstrating that the EMS and LMS medium formulations recapitulate lipid accumulation associated with the progression of MASLD.

Previous studies have shown that increased steatosis associated with MASLD progression impairs mitochondrial oxidative capacity, resulting in the increased production of reactive oxygen species (ROS) (Rolo et al., 2012; Florentino et al., 2024). Moreover, it has also recently been demonstrated that the PNPLA3 GG variant promotes the progression of MASLD by inducing mitochondrial dysfunction (Luukkonen et al., 2023; Gou et al., 2023; Martin-Fernandez et al., 2022). We next quantitatively assessed LPO in a 96-well plate format LAMPS (Miedel et al., 2019) constructed with the PNPLA3 GG variant or CC wild-type hepatocytes and nonparenchymal cells (NPCs) (LSECs, LX-2 cells, and THP-1 cells) that were maintained in NF, EMS, or LMS medium for 5 days (Supplementary Figure S6). At the conclusion of the experimental time course, the 96-well LAMPS was labeled with BODIPY 581/591 C11, a ratiometric fluorescent sensor for LPO, to determine the ratio of oxidized BODIPY (green) to reduced BODIPY (yellow), reflecting the overall LPO status within the model. Although a significant increase in the ratio of oxidized BODIPY/reduced BODIPY (OxBODIPY/ReBODIPY) was observed in the PNPLA3 GG variant 96-well plate LAMPS compared to the CC wild-type under each medium condition, demonstrating increased ROS production associated with the PNPLA3 GG variant (Supplementary Figure S6A,B), a significant increase in the ratio of OxBODIPY/ReBODIPY across medium conditions was only observed in the GG variant in the LMS medium compared to the NF medium (Supplementary Figure S6C). Taken together, these results are consistent with the lipid-handling defects that are associated with the PNPLA3 GG variant (Luukkonen et al., 2023; Gou et al., 2023; Martin-Fernandez et al., 2022).

### Increased production of pro-inflammatory cytokines is observed in the PNPLA3 rs738409 GG variant LAMPS compared to the CC wild-type LAMPS, demonstrating an increased pro-inflammatory environment

**FIGURE 3:**
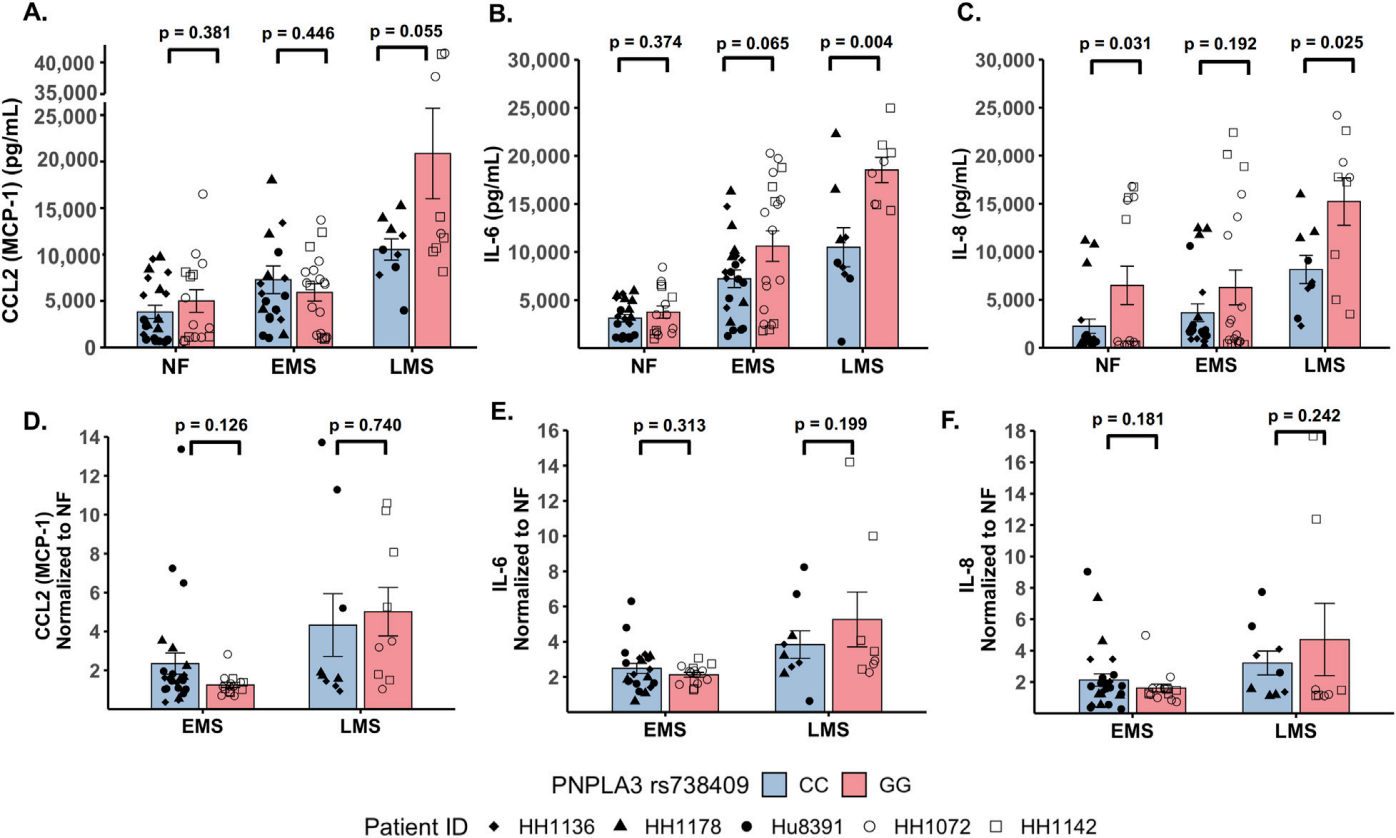
Increased pro-inflammatory cytokine production is observed in the PNPLA3 rs738409 GG variant compared to the CC wild-type LAMPS, demonstrating enhanced immune activation. (**A–C**: absolute cytokine values) For NF medium, a significant increase in IL-8 secretion **(C)** was observed in the PNPLA3 GG variant compared to the CC wild-type LAMPS. For the LMS medium, significant increases in the secretion of both IL-6 **(B)** and IL-8 **(C)** were observed in the PNPLA3 GG variant compared to the CC wild-type LAMPS, consistent with studies demonstrating increased immune activation and inflammation associated with the PNPLA3 GG variant (Kabbani et al., 2022; Krawczyk et al., 2020; Kirchmeyer et al., 2023). No significant differences in CCL2 secretion were observed between the PNPLA3 GG variant and CC wild-type LAMPS in any medium condition. (**D–F**: normalized to NF values) Absolute cytokine values shown in A–C were normalized to their respective NF control for each study. For normalized cytokine values, while no significant differences between the PNPLA3 GG and CC LAMPSs were observed in either EMS or LMS medium, the overall secretion levels of CCL2, IL-6, and IL-8 were all increased in EMS and LMS media compared to the NF medium. The statistical comparisons between medium types within each PNPLA3 genotype are shown in Supplementary Figure S7. Data were obtained on day 8 with a minimum of n = 3 LAMPSs from each patient ID for each medium condition. Plotted is the mean ± SEM. Statistical significance was assessed by ANOVA with Tukey’s test. *p*-value <0.05 was considered statistically significant.

The production of inflammatory cytokines is a significant factor contributing to the progression of MASLD (Duan et al., 2022; Ortiz-Lopez et al., 2022). Several experimental models demonstrate that the PNPLA3 rs738409 GG variant is associated with both elevated pro-inflammatory cytokine production and increased incidence of advanced fibrosis (Kostrzewski et al., 2020; Kabbani et al., 2022; Krawczyk et al., 2020). To investigate the effect of the PNPLA3 rs738409 GG variant on cytokine production, the PNPLA3 GG variant and CC wild-type LAMPS were maintained for 8 days in either NF, EMS, and LMS medium, and day-8 efflux samples were analyzed to measure the secretion of a panel of cytokines associated with MASLD progression, including CCL2, IL-6, and IL-8 (Braunersreuther et al., 2012; Fontes-Cal et al., 2021) (Figure 3; Supplementary Figure S7). Comparing absolute cytokine values between the PNPLA3 LAMPS in each medium type revealed no significant differences in CCL2 secretion between the PNPLA3 GG variant compared to the CC wild-type LAMPS under any medium condition (Figure 3A). A significant increase in IL-6 secretion was observed in the PNPLA3 GG variant compared to the CC wild-type variant in the LMS medium (Figure 3B), and a significant increase in IL-8 secretion was observed in the PNPLA3 GG variant compared to the CC wild-type LAMPS maintained in NF and LMS media (Figure 3C), suggesting a genotype-specific effect on cytokine secretion in the LMS medium.

In addition to the absolute cytokine values shown in Figures 3A–C, individual values were also normalized to their respective NF medium control from each individual study to determine the relative increases in individual cytokine production in EMS and LMS media (Figures 3D–F). Although no significant differences between the PNPLA3 GG and CC LAMPSs were observed in either EMS or LMS medium, the overall secretion levels of CCL2 (Figure 3D), IL-6 (Figure 3E), and IL-8 (Figure 3F) were all increased in EMS and LMS media compared to the NF medium. We also performed statistical comparisons between media types within each PNPLA3 genotype and showed that the secretion of all three cytokines was significantly higher in LMS compared to that in NF medium in both the PNPLA3 CC wild-type and GG variant LAMPSs (Supplementary Figure S7A–C). However, while all three cytokines were significantly increased in LMS compared to EMS medium in the PNPLA3 GG variant LAMPS, only IL-8 secretion was significantly increased in the PNPLA3 CC wild-type LAMPS in the LMS medium (Supplementary Figure S7A–C). Taken together, these results demonstrate that increased pro-inflammatory cytokine production is observed in the PNPLA3 GG variant compared to CC wild-type, consistent with the observed clinical phenotypes that are associated with this high-risk polymorphism (Valenti et al., 2010; Krawczyk et al., 2020; Singal et al., 2014; Shen et al., 2015; Park et al., 2023).

### Increased stellate cell activation and COL1A1 secretion is observed in the PNPLA3 rs738409 GG variant LAMPS compared to the CC wild-type LAMPS and is consistent with the increased incidence of advanced fibrosis in patients carrying the PNPLA3 high-risk allele

**FIGURE 4:**
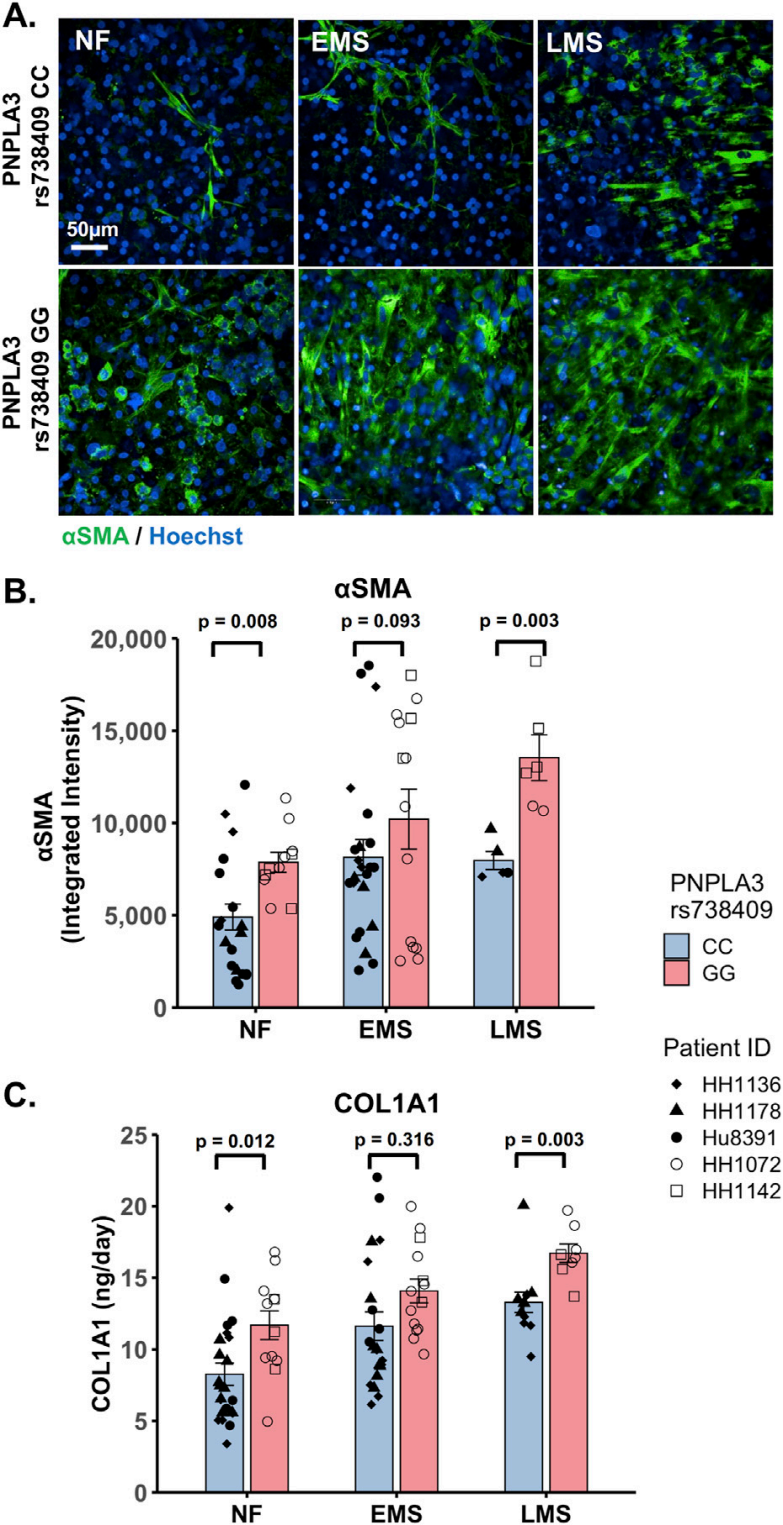
Increased stellate cell activation and COL1A1 secretion are observed in the PNPLA3 GG variant LAMPS compared to the CC wild-type LAMPS, demonstrating a more severe pro-fibrotic phenotype, consistent with the clinical characterization of the GG variant. **(A)** Representative images of the αSMA-labeled PNPLA3-LAMPS maintained in NF, EMS, and LMS media. Magnification: 40×; scale: 50 µm. **(B)** A significant increase in αSMA integrated intensity was observed in the PNPLA3 GG variant LAMPS compared to the CC wild-type LAMPS in both NF and LMS media, indicating an increase in stellate cell activation. **(C)** In addition, a significant increase in COL1A1 secretion was also observed in the PNPLA3 GG variant compared to the CC wild-type LAMPS in both NF and LMS media. Taken together, these results demonstrate that the PNPLA3 variant is associated with increased stellate cell activation and subsequent secretion of pro-fibrotic markers (COL1A1), consistent with the clinical characterization of this high-risk variant (Valenti et al., 2010; Krawczyk et al., 2020; Singal et al., 2014; Shen et al., 2015). Data were obtained on day 8 with a minimum of n = 3 LAMPSs from each patient lot for each condition and plotted as the mean ± SEM. Statistical significance was assessed by ANOVA with Tukey’s test. *p*-values <0.05 were considered statistically significant. Statistical analyses comparing across medium types for each PNPLA3 genotype were also performed and are shown in Supplementary Figure S8.

The PNPLA3 rs738409 GG variant is associated with an increased risk for MASLD progression and advanced fibrosis (Valenti et al., 2010; Krawczyk et al., 2020; Singal et al., 2014; Shen et al., 2015). We examined whether there were genotype-specific changes in stellate cell activation using fluorescence imaging to quantify the expression of αSMA, a marker for stellate cell activation, and production of the pro-fibrotic marker pro-collagen 1A1 (COL1A1) in the PNPLA3 GG variant and CC wild-type LAMPS that were maintained for 8 days in either NF, EMS, and LMS medium (Figure 4; Supplementary Figure S8). For both analyses, an overall increase in αSMA fluorescence intensity (Figures 4A,B) and secretion of COL1A1 (Figure 4C) was observed in the PNPLA3 GG variant LAMPS compared to the CC wild-type LAMPS in all three medium types, with significant increases observed in both NF and LMS media. In addition, comparisons across medium conditions within each PNPLA3 genotype demonstrated significant increases in αSMA fluorescence intensity under both EMS and LMS medium conditions compared to the NF medium for both the PNPLA3 CC wild-type and GG LAMPSs (Supplementary Figure S8A). Although the PNPLA3 CC wild-type LAMPS displayed a significant increase in COL1A1 secretion in both EMS and LMS media, COL1A1 secretion was significant only in the LMS medium for PNPLA3 GG LAMPS (Supplementary Figure S8B). Taken together, these results demonstrate that the presence of the GG variant results in an elevated pro-fibrotic state under both normal fasting and MASLD conditions, consistent with the role of this high-risk variant being associated with an increased risk for progression to advanced fibrosis (Valenti et al., 2010; Krawczyk et al., 2020; Singal et al., 2014; Shen et al., 2015).

### Both the PNPLA3 rs738409 GG variant LAMPS and CC wild-type LAMPS demonstrate overall excellent reproducibility for steatosis, pro-inflammatory cytokine secretion, and fibrosis metrics in NF and EMS media when the disease state, genotype, and patient cohort are segmented

Reproducibility was assessed for both the PNPLA3 GG and CC LAMPSs for LipidTOX (steatosis), normalized cytokine values (immune activation), and αSMA integrated intensity (stellate cell activation) for each patient cell lot used in the studies to generate Figures 1–4. The Numa Biosciences Eve Analytics™ platform was implemented to determine the reproducibility score for each metric for day-8 values (Schurdak et al., 2020; Miedel et al., 2024). Table 1 shows that excellent reproducibility was observed in both NF and EMS media except for CCL2 secretion in patient lot 8,391 among the PNPLA3 CC wild-type patient cell lots, as well as αSMA integrated intensity in patient lot 1,072 and IL-6 secretion in patient lot 1,142 among the PNPLA3 GG variant patient cell lots. Overall, this analysis demonstrates that the PNPLA3 LAMPS for both PNPLA3 genotypes is reproducible using a variety of MASLD-specific metrics in both NF and EMS media, while only one individual study was performed for the LMS medium using these patient cell lots, so reproducibility could not be assessed.

**TABLE 1:** PNPLA3-LAMPS demonstrate overall excellent reproducibility for steatosis, fibrosis, and immune activation when disease state, genotype and patient cohort are segmented.

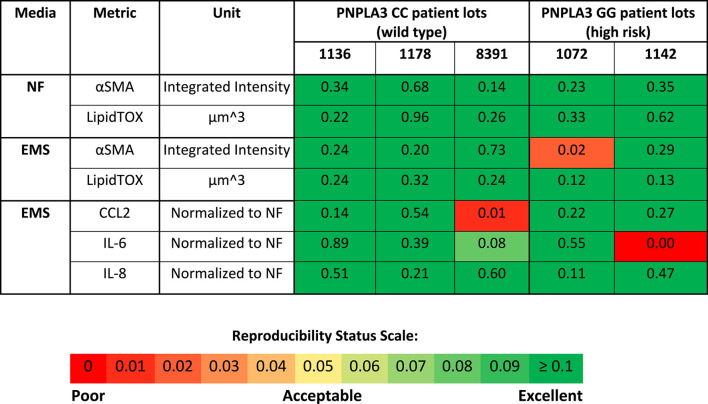

Reproducibility was assessed for αSMA integrated intensity, LipidTOX and normalized cytokine values for each patient lot used in the studies to generate Figures 1–4. Excellent reproducibility was observed for all media conditions except for αSMA integrated intensity in patient lot 1,072, CCL2 secretion in patient lot 8,391, and IL-6 secretion in patient lot 1,142. The reproducibility score is determined as described in the Materials and Methods section on the Day 8 measurement. Patient lot 8,391 was run in 6 independent studies, patient lot 1,072 was run in 3 independent studies except for the EMS condition where 4 studies were run, and patient lots 1,136, 1,178, and 1,142 were run in 2 independent studies. A *p*-value greater than 0.05 indicates that the means of the data points are similar (reproducible). The Reproducibility Status Scale displays *p*-value ranges from high (*p*-value >0.1) to low (*p*-value <0.05). Metadata and experimental data were captured in Numa Biosciences EveAnalytics™ platform (Miedel et al., 2024)

### Resmetirom treatment resulted in a greater reduction in steatosis in the PNPLA3 CC wild-type LAMPS compared to the GG variant LAMPS, demonstrating genotype-specific inhibition of MASLD progression

Overall, the role of the PNPLA3 rs738409 variant in the response to MASLD drug treatment remains uncertain (Wang et al., 2018). Using the same approach outlined in Supplementary Figure S1 to examine the effect of the PNPLA3 GG variant on MASLD disease progression, we next used both the PNPLA3 GG variant and CC wild-type LAMPS to evaluate the efficacy of resmetirom (Rezdiffra™), a thyroid hormone receptor beta (THR-β) agonist recently approved for the treatment of MASLD (Harrison et al., 2024; Harrison et al., 2019; Harrison et al., 2023). We determined that no detectable amount of resmetirom was adsorbed by the PDMS component of the LAMPS device (Supplementary Table S3). The PNPLA3 GG variant and CC wild-type LAMPS were maintained for 8 days in the EMS medium containing resmetirom (1 μM) or vehicle control (Supplementary Figure S1C). The concentration of 1 µM chosen for these studies is the approximate reported Cmax for resmetirom (0.9 µM) (Taub et al., 2013). The effect of resmetirom on disease progression was evaluated using a panel of metrics, including steatosis, pro-inflammatory cytokine secretion, stellate cell activation, and fibrosis.

To evaluate LAMPS functionality and cytotoxicity under conditions of resmetirom treatment, efflux samples were collected on days 2, 4, 6, and 8 from the PNPLA3 GG variant and CC wild-type LAMPS to measure the secretion of ALB and BUN for LAMPS functionality and LDH for LAMPS cytotoxicity (Supplementary Figure S9). In both the PNPLA3 GG variant and CC wild-type LAMPS, no significant changes were observed between resmetirom treatment and vehicle control for the secretion of ALB (Supplementary Figure S9A,D), BUN (Supplementary Figure S9B,E), or LDH (Supplementary Figure S9C,F), indicating that resmetirom does not adversely affect model functionality or cytotoxicity (Harrison et al., 2024; Harrison et al., 2023).

**FIGURE 5:**
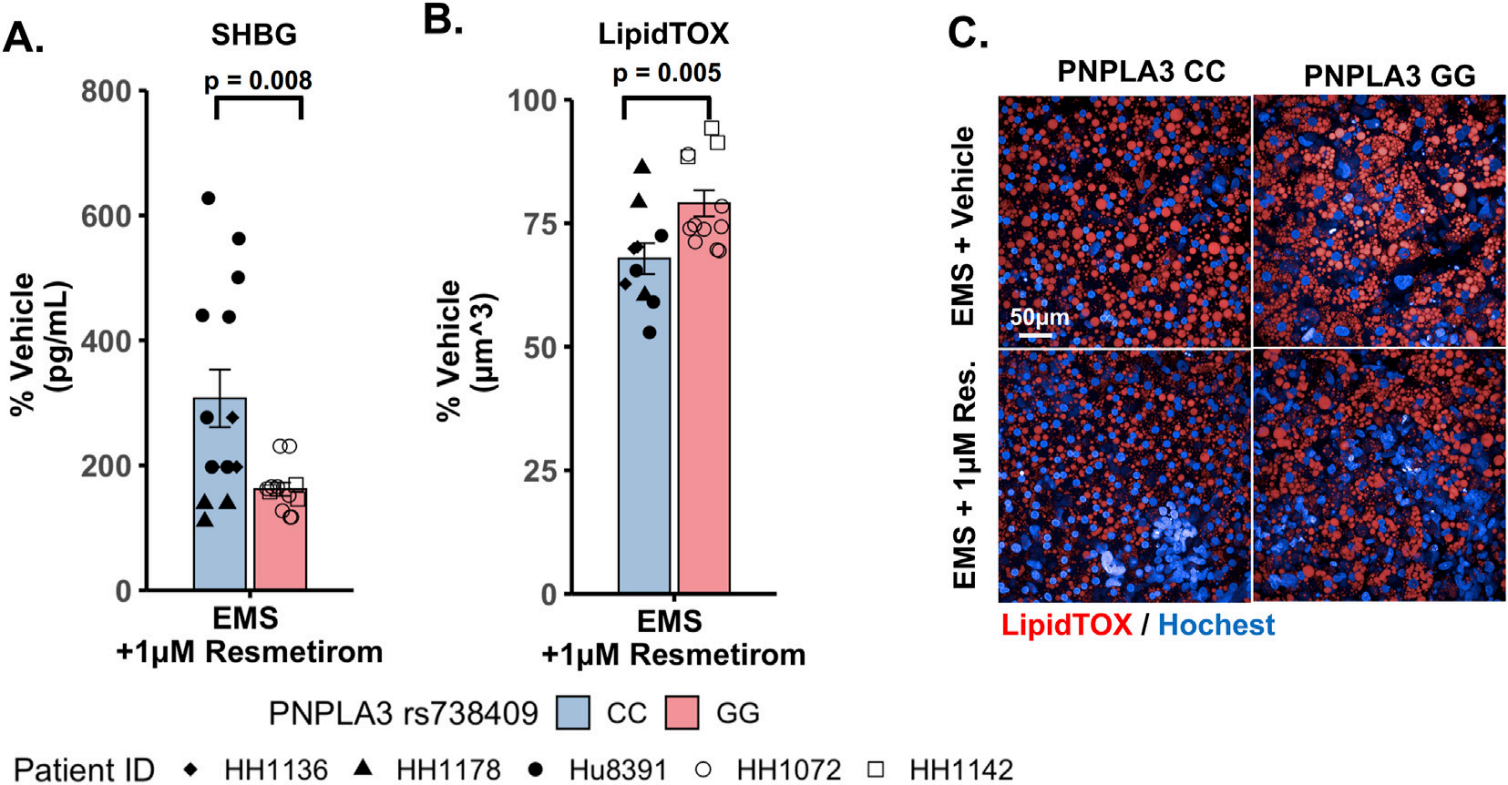
Resmetirom treatment resulted in a greater reduction in steatosis in the PNPLA3 CC wild-type LAMPS than that in the GG variant LAMPS, demonstrating genotype-specific inhibition of MASLD progression. PNPLA3 LAMPS models were maintained for 8 days in the EMS medium containing resmetirom (1 μM) or DMSO vehicle control. The secretion of the sex hormone-binding globulin (SHBG) was assessed to determine the on-target pharmacodynamic activity of resmetirom, and LipidTOX staining was quantified to determine the effect of resmetirom on steatosis. **(A)** Treatment with 1 µM resmetirom increased SHBG secretion in both PNPLA3 LAMPSs, demonstrating the on-target pharmacodynamic activity of resmetirom. However, the PNPLA3 CC wild-type LAMPS displayed significantly higher SHBG secretion than the GG variant LAMPS. **(B)** Resmetirom treatment resulted in a significantly greater reduction in steatosis in the PNPLA3 CC wild-type LAMPS (∼33%) compared to the PNPLA3 GG variant (∼20%). **(C)** Representative images of LipidTOX-labeled PNPLA3 LAMPS maintained in the EMS medium with and without 1 µM resmetirom. Magnification: 40×; scale: 50 µm. Data were obtained on day 8 with n = 3 LAMPSs from each patient lot for each condition and were plotted as the average % vehicle ± SEM. Statistical significance was assessed by ANOVA with Tukey’s test. *p*-values <0.05 were considered statistically significant. Statistical analysis comparing resmetirom treatment with the vehicle control within each PNPLA3 genotype was performed, as shown in Supplementary Figure S10.

We evaluated the on-target pharmacodynamic activity of resmetirom by quantifying the secretion of SHBG in the PNPLA3 GG variant and CC wild-type LAMPS treated with 1 µM resmetirom as the SHBG is a direct transcriptional target of THR-β activity in the liver (Harrison et al., 2023; Karim and Bansal, 2023). On day 8, resmetirom treatment resulted in significantly increased SHBG secretion in both the GG variant and CC wild-type LAMPSs (Supplementary Figure S10A), supporting the on-target pharmacodynamic activity of resmetirom. In addition, the PNPLA3 CC wild-type LAMPS displayed significantly higher SHBG secretion compared to the GG variant LAMPS (Figure 5A). We then evaluated genotype-specific effects on steatosis using fluorescence imaging to quantify LipidTOX staining in the PNPLA3 GG variant and CC wild-type LAMPS that were maintained for 8 days in EMS-containing resmetirom (1 μM) or vehicle control. Although treatment with resmetirom significantly reduced steatosis in both the PNPLA3 GG variant and CC wild-type LAMPSs compared to their respective vehicle controls (Supplementary Figure S10B), a significantly greater reduction in steatosis was observed in the PNPLA3 CC wild-type LAMPS (∼33%) compared to the PNPLA3 GG variant (∼20%) (Figures 5B,C), indicating that resmetirom has better efficacy in the PNPLA3 CC wild-type LAMPS.

### Resmetirom treatment significantly reduced the secretion of the pro-inflammatory cytokine IL-6 in the PNPLA3 GG variant LAMPS compared to the CC wild-type LAMPS

**FIGURE 6:**
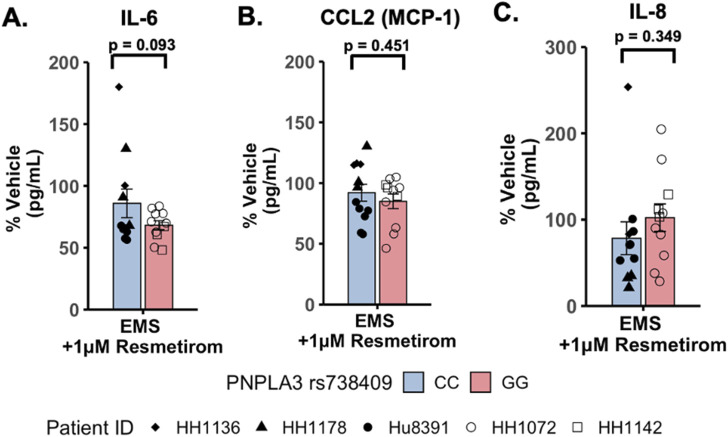
Resmetirom treatment did not result in any significant changes in pro-inflammatory reduction between the PNPLA3 GG variant and CC wild-type LAMPS. No significant differences in the reduction (% of vehicle control) in IL-6 **(A)**, CCL2 **(B)**, or IL-8 **(C)** were observed between the PNPLA3 GG variant and CC wild-type LAMPS upon treatment with 1 µM resmetirom. Data (% of vehicle control) were obtained on day 8 with a minimum of n = 3 LAMPSs from each patient lot for each condition and are plotted as ± SEM. Statistical significance was assessed by ANOVA with Tukey’s test. *p*-values <0.05 were considered statistically significant.

To evaluate potential differences in cytokine secretion in response to resmetirom treatment, the PNPLA3 GG variant and CC wild-type LAMPSs were maintained for 8 days in the EMS medium containing resmetirom (1 μM) or vehicle control, and day-8 efflux samples were analyzed to measure the secretion of IL-6, CCL2, and IL-8. When the reduction in individual secreted cytokines (% of vehicle control) was compared directly between the PNPLA3 GG variant and CC wild-type LAMPSs, no genotype-specific differences were observed (Figures 6A–C). However, resmetirom treatment significantly reduced IL-6 secretion compared to the vehicle control in the PNPLA3 GG variant LAMPS (∼30% reduction) but not in the PNPLA3 CC wild-type LAMPS (∼15% reduction) (Supplementary Figure S11A), while no reduction in either CCL2 (Supplementary Figure S11B) or IL-8 (Supplementary Figure S11C) was observed for either PNPLA3 LAMPS genotypes relative to the vehicle control. These results suggest that resmetirom has a larger effect on IL-6 secretion in the PNPLA3 GG variant.

### Resmetirom treatment resulted in a greater reduction in stellate cell activation and COL1A1 secretion in the PNPLA3 CC wild-type LAMPS compared to the GG variant LAMPS, demonstrating genotype-specific inhibition of MASLD progression

**FIGURE 7:**
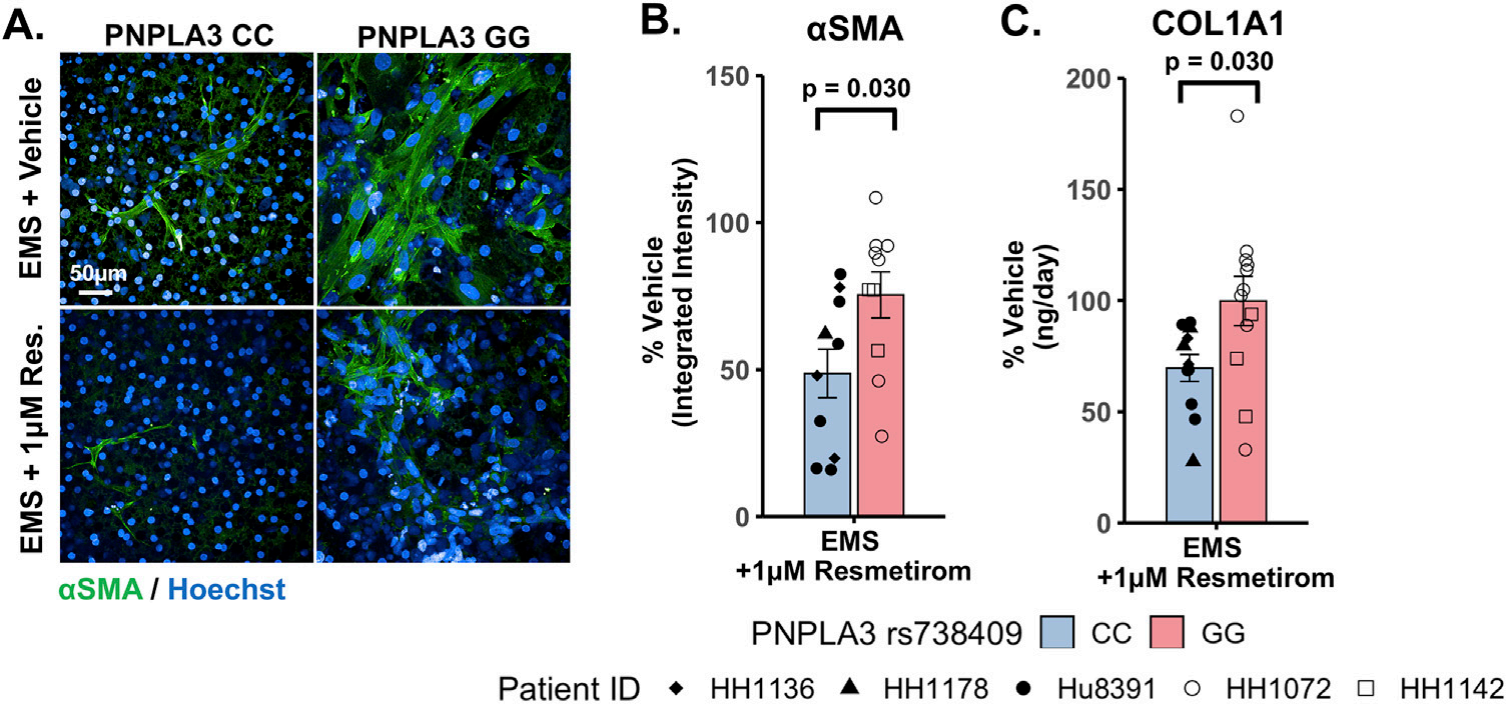
Resmetirom treatment resulted in a greater reduction in stellate cell activation and COL1A1 secretion in the PNPLA3 CC wild-type LAMPS than that in the GG variant LAMPS. **(A)** Representative images of the αSMA-labeled PNPLA3 LAMPS maintained in the EMS medium with 1 µM resmetirom or vehicle control. Magnification: 40×; scale: 50 µm. **(B,C)** A significantly greater reduction in both αSMA integrated intensity (B; 50% vs 25%) and the secretion of COL1A1 (C; 35% vs 0%) was observed in the PNPLA3 CC wild-type LAMPS compared to the PNPLA3 GG variant LAMPS. Data were obtained on day 8 with n = 3 LAMPSs from each patient lot for each condition and were plotted as the average % vehicle ± SEM. Statistical significance was assessed by ANOVA with Tukey’s test. *p*-values <0.05 were considered statistically significant. Statistical analysis comparing resmetirom treatment with the vehicle control within each genotype was performed, as shown in Supplementary Figure S12.

To evaluate genotype-specific differences in resmetirom treatment on the progression of pro-fibrotic phenotypes in the PNPLA3 GG variant and CC wild-type LAMPSs, we quantified stellate cell activation (αSMA expression) using fluorescence imaging microscopy and pro-fibrotic marker secretion (COL1A1) in the PNPLA3 LAMPS maintained for 8 days in the EMS medium containing resmetirom (1 μM) or vehicle control. Resmetirom significantly reduced both αSMA integrated intensity (Supplementary Figure S12A) and the secretion of COL1A1 (Supplementary Figure S12B) compared to the vehicle control in the PNPLA3 CC wild-type LAMPS but not the GG variant LAMPS. In addition, a significantly greater reduction in both αSMA integrated intensity (50% vs 25%) (Figures 7A,B) and the secretion of COL1A1 (35% vs 0%) (Figure 7C) was observed in the PNPLA3 CC wild-type LAMPS than that in the PNPLA3 GG variant, demonstrating better resmetirom efficacy for both stellate cell activation and production of pro-fibrotic markers.

## Discussion

The clinical variability and pathophysiologic heterogeneity of MASLD have contributed to the challenges in the therapeutic development of this disorder. To begin to address this challenge, we initiated a MASLD precision medicine approach that harnesses the use of the LAMPS constructed with primary hepatocytes genotyped for the wild-type PNPLA3 and the MASLD-associated genetic variant PNPLA3 rs738409. As an initial step in this process, our goal was to establish the disease progression, severity, and response to drug treatment in a primary cell-focused LAMPS, where fully adult hepatocytes and LSECs are combined with well-characterized human cell lines for stellate and Kupffer cells (Gough et al., 2021; Lefever et al., 2022; Saydmohammed et al., 2021; Lee-Montiel et al., 2017; Miedel et al., 2019; Vernetti et al., 2016) that will serve as a critical reference for evaluating the functionality, disease characteristics, and response to the drug using the MPS constructed from patient-derived iPSCs.

We showed here that the LAMPS model recapitulates key phenotypes of early and late MASLD, along with key differences in the response to drug treatment in the LAMPS constructed with either wild-type or high-risk PNPLA3 alleles. Previous RNAseq studies in the LAMPS using these medium formulations suggest that although the different media can induce an accelerated disease progression, the clinical pathophysiological mechanisms appear to be preserved in the MPS. To quantitatively interpret the data on disease progression and response to drug treatment, the model had to be reproducible (Sakolish et al., 2021b; Gough et al., 2016; Schurdak et al., 2020; Miedel et al., 2024). The LAMPS demonstrated overall excellent reproducibility for steatosis, fibrosis, and cytokine production when disease-state medium formulation, genotype, and patient hepatocyte lots were segmented (Table 1). Our results demonstrating differences in the PNPLA3 CC wild-type compared to the GG LAMPS are consistent with those of clinical studies in humans (Eslam et al., 2018; Dongiovanni et al., 2013; Krawczyk et al., 2020). The high-risk PNPLA3 GG variant LAMPS demonstrated increased steatosis, pro-inflammatory cytokine secretion, stellate cell activation, and secretion of the pro-fibrotic marker COL1A1 (Table 2), consistent with the clinical characterization of this polymorphism. In addition, we observed genotype-specific differences in response to drug treatment as resmetirom demonstrated greater efficacy in the PNPLA3 wild-type CC LAMPS than that in the GG variant in the reduction of steatosis, stellate cell activation, and the secretion of marker COL1A1 (Table 3).

**TABLE 2:** A summary of the MASLD progression in PNPLA3 CC wild type and GG variant LAMPS normalized to PNPLA3 CC wild type in NF.

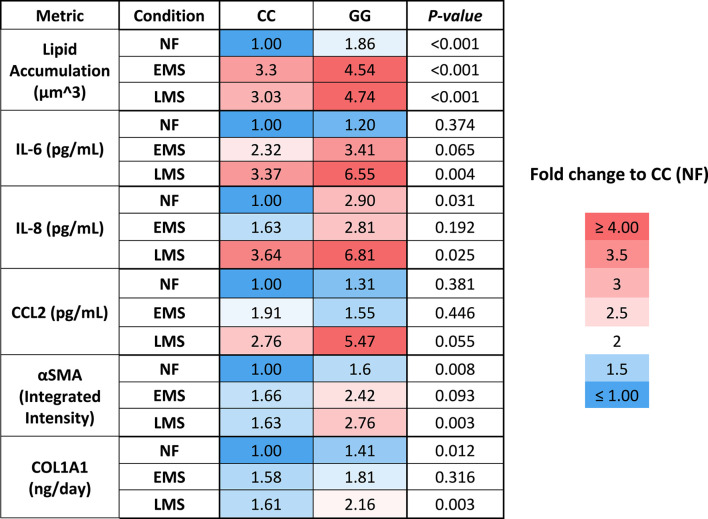

**TABLE 3:** A summary of genotype-specific response to resmetirom in PNPLA3 CC wild type and GG variant LAMPS.

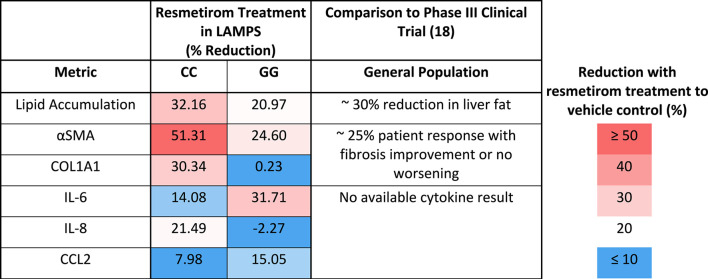

Reduction with resmetirom treatment to vehicle control (%)

The PNPLA3 polymorphism is associated with increased susceptibility to MASLD, disease progression to cirrhosis, and risk of developing HCC, and prior research supports the incorporation of the PNPLA3 genotype into prognostic scores for predicting the risk of disease progression (Kabbani et al., 2022; Krawczyk et al., 2020). Our experimental results demonstrate that the PNPLA3 rs738409 GG variant LAMPS exhibits increased steatosis in NF, EMS, and LMS media compared to the wild-type CC LAMPS, consistent with the clinical characterization describing abnormal lipid homeostasis in these patients (Romeo et al., 2008; Mann and Anstee, 2017; Mann et al., 2020; Luukkonen et al., 2023; Luukkonen et al., 2019). Our cytokine secretion data showed an increased production of IL-6 and IL-8 in the PNPLA3 GG LAMPS compared to the wild-type CC LAMPS in LMS medium, which is consistent with recent *in vivo* and *in vitro* studies (Kostrzewski et al., 2020; Kabbani et al., 2022). However, these experimental data conflict with a recent clinical study that examined serum cytokine levels in a cohort of 123 genotyped patients, finding no significant impact of PNPLA3 polymorphism on cytokine levels within this cohort (Kirchmeyer et al., 2023). Although, overall, our data from this study largely agree with the published clinical characterization of the PNPLA3 GG high-risk allele (steatosis, stellate cell activation, and fibrotic marker production), larger clinical studies, as well as further *in vivo* and *in vitro* studies, are required to fully determine whether cytokine profiles are impacted by the GG variant. These studies are critical because they will serve to both further validate that *in vitro* MPS platforms recapitulate actual clinical patient data and evaluate whether differences in the production of specific cytokines can be used as potential biomarkers for stratifying MASLD patients with or without the PNPLA3 polymorphism.

An overarching goal of our research is to develop a qualified human liver MPS drug discovery tool (DDT) to address several important contexts of use (CoUs), including toxicology, drug discovery/profiling, and clinical trials on chips. An immediate question is whether the MASLD LAMPS model could be used as a tool for segmenting patient cohorts based on the response to approved drugs, drug candidates (before or after entering the clinic or failed due to missing endpoints in general patient populations), and predicted drugs for repurposing (Gough et al., 2021; Lefever et al., 2022). Our strategy was to first focus on the early stages of MASLD progression with stage 3 or less fibrosis that is maintained in the EMS medium. Our first step was to determine whether the PNPLA3 GG variant LAMPS and wild-type CC LAMPS in the EMS “lifestyle” state would respond distinctly to the recently approved drug, resmetirom, a liver-targeted THR-β-selective agonist designed to target causes of MASH with moderate-to-advanced liver fibrosis (Harrison et al., 2024; Harrison et al., 2019; Harrison et al., 2023). The initial study reported here was to determine the level of inhibition of disease progression over 8 days with a single dose (1 µM) based on the validated panel of metrics, including steatosis, pro-inflammatory cytokine secretion, and early fibrosis (stellate cell activation and secretion of COL1A1). Overall, we observed genotype-specific differences in the response to resmetirom treatment that were consistent with a recent clinical study where ∼15% of a general population in phase III exhibited improvement in fibrosis or no worsening with resmetirom treatment (Harrison et al., 2024). We did observe significantly less SHBG secretion in the GG variant LAMPS compared to the CC wild-type LAMPS upon treatment with resmetirom (Figure 5A), suggesting less overall on-target pharmacodynamic activity of resmetirom in the GG variant LAMPS. We observed genotype-specific differences in response to resmetirom consistent with this, demonstrating that steatosis, stellate cell activation, and secretion of the pro-fibrotic marker COL1A1 were significantly reduced in the PNPLA3 wild-type LAMPS compared to the GG LAMPS. However, in contrast to these results, we also observed a greater decrease in IL-6 secretion in the GG LAMPS compared to the CC wild-type LAMPS, which is contradictory to our SHBG secretion data on CC and GG LAMPSs. IL-6 was the only metric that did not align with what has been recently published for the clinical evaluation of resmetirom, and the reason for this discrepancy remains unclear, warranting additional experimental investigation. Although the mechanism of action for THR-β agonists includes the stimulation of β-oxidation, reduced production and secretion of very low-density lipoprotein, and the reduction in hepatic triglycerides, more work is required to obtain a better understanding of how IL-6 regulation may differ in the PNPLA3 GG genetic background compared to the wild type and how this results in an enhanced effect for treatment with THR-β agonists like resmetirom. In addition, the interplay between multiple THR-β-expressing liver cell types is also important to consider regarding the response to resmetirom as other liver cell types have been shown to express THR-β (Saponaro et al., 2020). THR-β-specific agonists like resmetirom are potent lipid-lowering drugs; however, significant improvement in fibrosis is also observed upon treatment with resmetirom. Thus, more mechanistic studies are required to discern the underpinnings of how the anti-fibrotic effects of THR-β agonists occur. Now that we have established a benchmark *in vitro* response to resmetirom that directly leads to a hypothesis that can be tested by retrospective clinical analysis, we can extend these studies using single-cell analysis to investigate dynamic heterotypic cell interactions.

Our study has several strengths, including the use of genotyped primary hepatocytes that serve as an important benchmark for future studies using the MPS constructed with iPSC-derived liver cells from patients. A critical question in using iPSC-derived cells as a precision medicine tool has been the level of overall maturity and functionality, and this requires a benchmark. Another strength of our study is that it demonstrates genotype-specific differences in both MASLD progression and response to drug treatment, indicating that a reproducible patient-derived MPS could be harnessed to identify disease-state-specific biomarkers that would optimize patient subgroups for therapeutic testing strategies. One limitation to this study was that we tested two patient lots of PNPLA3 GG variant hepatocytes in the LAMPSs that were constructed with non-isogenic NPCs. Thus, the present work serves as a starting point using a hybrid MPS configuration toward our overall effort toward constructing the MPS using iPSCs from individual patients (patient biomimetic twins). Second, the addition of resmetirom to the EMS medium in our present study occurred at the outset of establishing a flow in the LAMPS to test the effect of resmetirom on preventing disease progression. Future studies using the existing hybrid LAMPS model, while we validate the PBTs, will evaluate the effect of resmetirom on reversing MASLD phenotypes in the LAMPS where EMS disease features have been established. Additional steps will include full dose–response curves on a larger genotyped pool of patient backgrounds, including PNPLA3 and other known MASLD-associated variants. Individual drug candidates or combinations of drugs after the MASLD LAMPSs have been progressed to EMS, and LMS “lifestyle states” will be tested to explore whether later stages of disease progression can be halted and/or reversed. Furthermore, the present model and the future iPSC-derived MPS will play a critical role in defining the mechanisms of action of drug candidates and drugs.

In summary, there are many efforts toward developing precision medicine platforms for MASLD, including a variety of MPS designs, organoids, and humanized murine models. In addition, a combination of small molecules, antisense oligonucleotides, and siRNA-based silencing of other MASLD-relevant genes and targets are under investigation (Fraile et al., 2021; Mak et al., 2023). Looking forward, we will continue the characterization of iPSC-derived liver cells from patients who have been enrolled in the University of Pittsburgh Medical Center Liver Steatosis and Metabolic Wellness Clinic and their incorporation into PBTs (Figure 1) (Gough et al., 2021; Florentino et al., 2024; Faccioli et al., 2023; Florentino et al., 2022). The reproducibility, functionality, and response to disease progression and drug treatments will be benchmarked to the primary cell-focused study reported here. Thus, the present work serves as a starting point using a hybrid MPS configuration toward our overall effort toward constructing an MPS using iPSCs from individual patients (PBTs) that has potential implications for precision risk stratification, enrichment of patient cohorts for clinical trials, and selection of approved therapies for the management of patient subgroups with MASLD.

We have patient clinomics data and are beginning to develop a variety of omics data on selected patients for clinical reference and future computational modeling (patient digital twins [PDTs]). The goal is to produce cohorts of PBTs where the genotype, lifestyle, and environment histories, as well as co-morbidities, are known. Having specific cohorts of patients and their PBTs representing some of the major sources of MASLD patient heterogeneity will facilitate the development of advanced qualified liver MPS DDTs with many CoUs that will aid drug discovery and development, including optimized clinical trials on chips by the selection of high-probability responders before testing in patients. Based on a growing set of clinomics and omics datasets on the enrolled patients, we will be in a position to extend our combined human MPS and quantitative system pharmacology strategy (Taylor et al., 2019; Stern et al., 2016) to include the development of PDTs to complement the PBTs for predicting patient cohort-specific toxicity and efficacy. We project that the integration of PDTs and PBTs for MASLD and other diseases will play critical roles in the advancement of precision medicine efforts.

## Data Availability

The raw data supporting the conclusion of this article will be made available by the authors, without undue reservation.
