# Supplementary material for: Comparison of wild-type and high-risk PNPLA3 variants in a human biomimetic liver microphysiology system for metabolic dysfunction-associated steatotic liver disease precision therapy
Source: Front Cell Dev Biol. 2024 Sep 11;12:1423936. doi: 10.3389/fcell.2024.1423936 (PMC11422722; doi:10.3389/fcell.2024.1423936)
Supplement: Supplementary file 1 [file Presentation1.pdf]

Supplementary Material

Comparison of Wild-Type and High-risk PNPLA3 variants in a Human Biomimetic Liver Microphysiology System for Metabolic Dysfunction-associated Steatotic Liver Disease Precision Therapy

Mengying Xia[1, *], Mahboubeh Varmazyad[1, *], Iris Pla-Palacín[1, *], Dillon C. Gavlock[1], Richard DeBiasio[1], Gregory LaRocca[1], Celeste Reese[1], Rodrigo Florentino[2,3], Lanuza A.P. Faccioli[2,3], Jacquelyn A. Brown[1,5], Lawrence A. Vernetti[1,5], Mark Schurdak[1,4, 5], Andrew M. Stern[1,5], Albert Gough[1,4], Jaideep Behari[4,6], Alejandro Soto-Gutierrez[1,2,3,4], D. Lansing Taylor[1,4,5,+] and Mark T. Miedel[1,2,4,+ #]

[1]Drug Discovery Institute, University of Pittsburgh, Pittsburgh, PA 15261, USA;
[2]Department of Pathology, School of Medicine, University of Pittsburgh, Pittsburgh, PA 15261, USA
[3]Center for Transcriptional Medicine, Pittsburgh Liver Research Center, University of Pittsburgh, Pittsburgh, PA 15261, USA;
[4]Pittsburgh Liver Research Center, University of Pittsburgh, Pittsburgh, PA 15261, USA;
[5]Department of Computational and System Biology, School of Medicine, University of Pittsburgh, PA 15260, USA
[6]Division of Gastroenterology, Hepatology and Nutrition, School of Medicine, University of Pittsburgh, Pittsburgh, PA 15261, USA

***Co-first authors, + co-senior authors, # corresponding author**

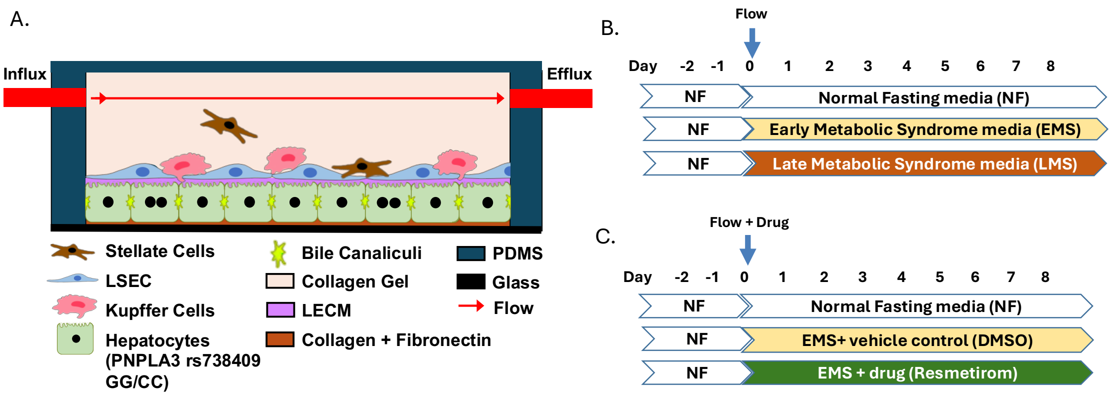

Figure S1. Overview of the experimental setup for LAMPS MASLD disease progression and drug testing studies. (A) LAMPS were constructed with 4 liver cell types sequentially layered starting with primary hepatocytes (PNPLA3 rs738409 GG/CC) followed by the addition of non-parenchymal cells (NPCs) including primary liver sinusoidal endothelial cells (LSECs), and LX-2 (hepatic stellate cells) and activated THP-1 (Kupffer-like cells) cell lines as previously described (1-5). (B-C) Experimental timeline used to monitor disease progression and drug testing in LAMPS using the previously described media formulations shown in Table S1 (NF, EMS, and LMS). For these studies, LAMPS were maintained at a flow rate of 5 µL/h over an 8-day time course to mimic zone 3 oxygen tension (2). For drug testing studies, resmetirom was added to EMS medium at time 0 and compared to a 0.02% DMSO vehicle control to evaluate the efficacy of this drug on MASLD progression.

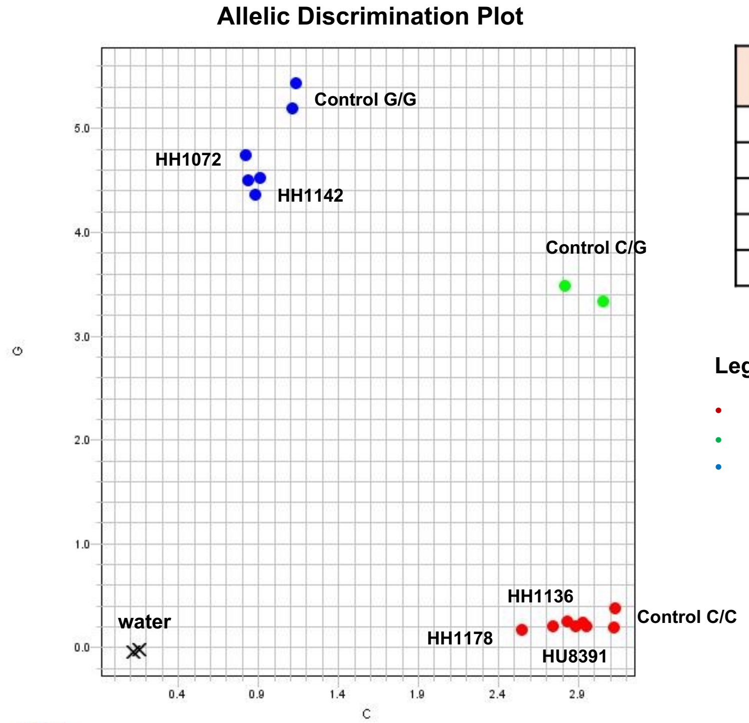

Patient ID	PNPLA3 rs738409
HH1142	GG
HH1072	GG
HH1136	CC
HH1178	CC
HU8391	CC

Legend

- Homozygous C/C
- Heterozygous C/G
- Homozygous G/G

Figure S2. Genotyping analysis of primary hepatocyte cell lines used in this study demonstrate both the PNPLA3 CC wild type and GG high-risk variant genotypes. Genotyping of the primary hepatocyte lots used in these studies was performed by extracting genomic DNA from each patient cell lot, and genotyping for the PNPLA3 rs738409 allele was then performed using real-time PCR. As shown in the allelic discrimination plot, hepatocyte lots HH136, HH178, HU8391 contained the CC PNPLA3 wild type allele, while hepatocyte lots HH142 and HH1072 contained the GG high-risk variant allele.

Table S1. The formulations of NF, EMS and LMS media drive MASLD progression based on the effect of lifestyle on blood chemistry.

Medium Component	NF (Normal Fasting)	EMS (Early Metabolic Syndrome; MASLD)	LMS (Late Metabolic Syndrome; MASH, T2D)
**Glucose**	5.5 mM	11.5 mM	20 mM
**Insulin**	10 pM	10 nM	10 nM
**Glucagon**	100 pM	10 pM	10 pM
**Oleic acid**	-	200 µM	200 µM
**Palmitic acid**	-	100 µM	100 µM
**Lipopolysaccharide (LPS)**	-	-	0.25 µg/mL
**Transforming Growth Factor β1 (TGF-β1)**	-	-	5 ng/mL

Media formulations were designed to mimic disease progression from the normal fasting (NF) to early metabolic syndrome (EMS; MASLD) and late metabolic syndrome (LMS; MASH, T2D) state. These media formulations were developed using glucose-free Williams E base medium supplemented with physiologically relevant levels of glucose, insulin, glucagon, oleic acid, palmitic acid and molecular drivers of fibrosis including TGF-β1 and LPS (4, 6-8).

Table S2: Clinical characteristics of PNPLA3 rs738409 GG variant and CC wild type primary hepatocytes used in LAMPS studies.

Vendor	Lot ID	Genotype (PNPLA3 rs738409)	Age	Sex	Race	BMI	Smoking	Alcohol	Infectious diseases*
**DLS**	HH1072	GG	40	Female	Caucasian	37.3	Yes	Yes	HBV-, HCV-, HIV-, CMV-
**DLS**	HH1142	GG	27	Female	Caucasian	25	No	No	HBV-, HCV-, HIV-, CMV+
**DLS**	HH1178	CC	66	Female	Caucasian	31.6	Yes	No	HBV-, HCV-, HIV-, CMV+
**DLS**	HH1136	CC	N/A	Male	Caucasian	27.34	N/A	N/A	N/A
**Thermo Fisher**	Hu8391	CC	62	Male	Caucasian	22.2	Yes	Yes	HBV-, HCV-, HIV-, CMV-

*Discovery Life Sciences, DLS; Hepatitis B Virus, HBV; Hepatitis C Virus, HCV; Human Immunodeficiency Virus, HIV; Cytomegalovirus, CMV.

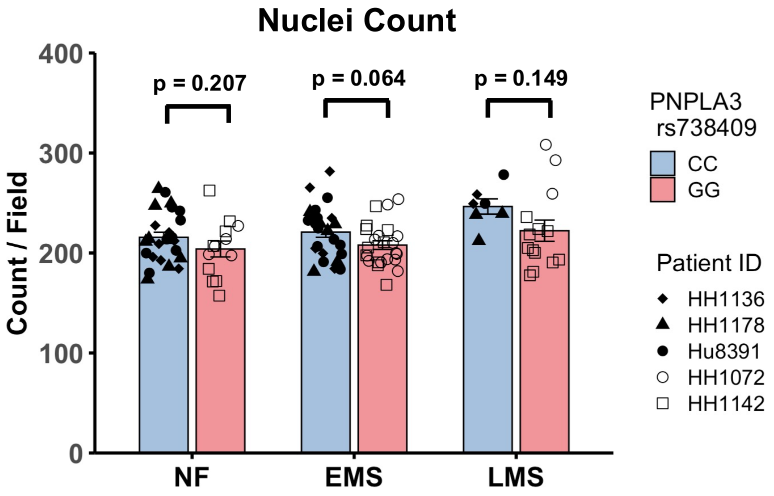

Figure S3. Similar cell counts were observed in both PNPLA3 rs738409 GG variant and CC wild type LAMPS in each media condition. No significant difference was observed in nuclei count between the two PNPLA3 genotypes in LAMPS maintained in each condition. Cell counts were obtained on Day 8 by identifying and counting the number of Hoechst-positive objects per field with a minimum of n = 3 LAMPS from each patient lot for each media condition and plotted mean ± SEM. Statistical significance was assessed by ANOVA with Tukey's test. p-value < 0.05 was considered statistically significant.

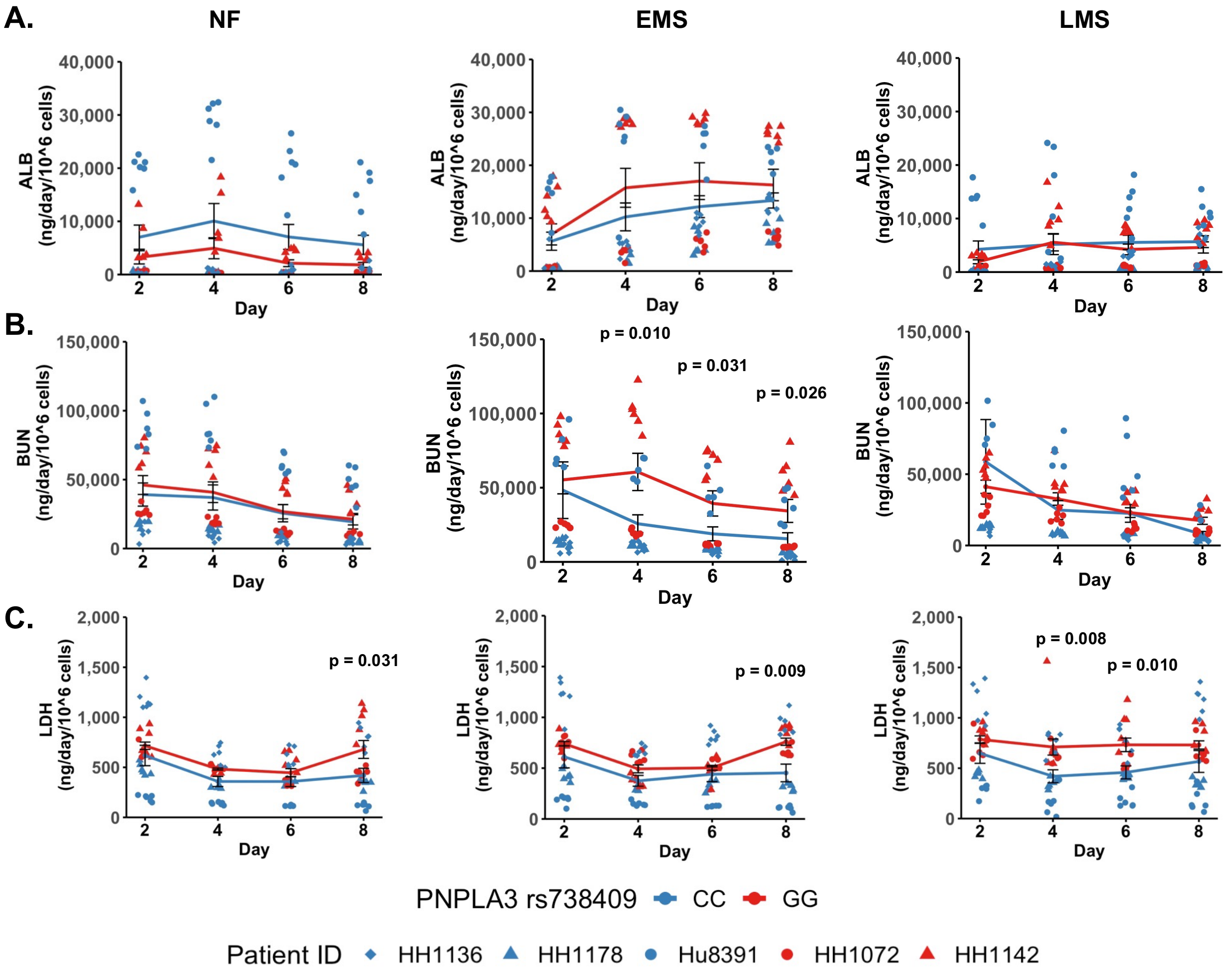

Figure S4. While PNPLA3 rs738409 GG variant and CC wild type LAMPS exhibited similar overall model functionality, PNPLA3 GG LAMPS displayed increased cytotoxicity across media types. (A-C) The secretion of albumin (ALB; A), blood urea nitrogen (BUN; B), and lactate dehydrogenase (LDH; C) were compared over the 8-day time course in PNPLA3 GG variant and CC wild type LAMPS to identify genotype-specific differences in LAMPS functionality (ALB and BUN) and cytotoxicity (LDH) in each media type (NF, EMS, LMS). While no significant changes in ALB secretion (A) were observed between PNPLA3 GG variant and CC wild type LAMPS, indicating similar overall model functionality, a significant increase in BUN secretion (B) was observed on days 4, 6 and 8 in EMS medium in PNPLA3 GG LAMPS. (C) A significant increase in LDH secretion was observed in both NF and EMS media on day 8, and on days 4 and 6 in LMS medium, suggesting an overall increase cytotoxicity in PNPLA3 GG LAMPS, consistent with its characterization as a high-risk variant. Data were plotted mean ± SEM from a minimum of n = 3 LAMPS from each patient lot for each media condition. Statistical significance was assessed by ANOVA with Tukey's test for each indicated time point. Only p-values < 0.05 were considered statistically significant and are indicated.

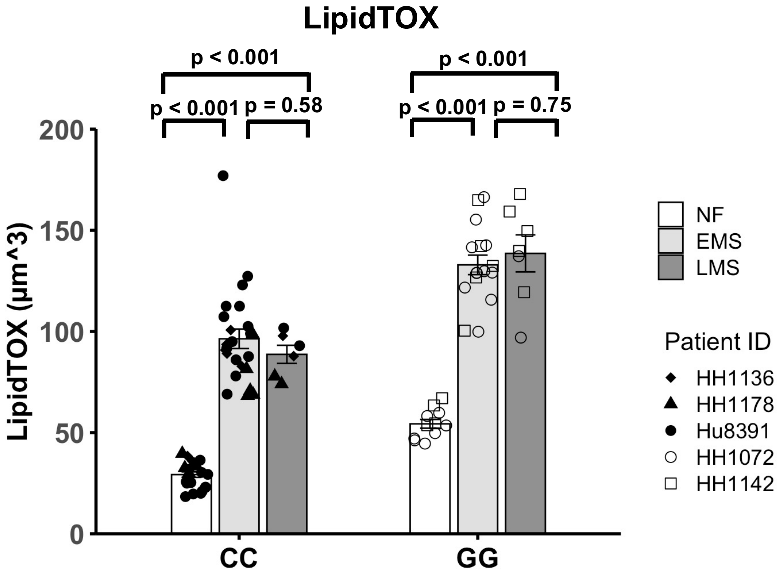

Figure S5. Increased steatosis was observed in both EMS and LMS media compared to NF medium in both PNPLA3 CC and GG LAMPS, demonstrating lifestyle induced MASLD progression. Steatosis was quantified by quantitative fluorescence imaging of LipidTOX labeled samples in each media condition. Significant increases in steatosis were observed in both EMS and LMS media compared to NF medium within each PNPLA3 genotype. Data were obtained on Day 8 with a minimum of n = 3 LAMPS from each patient lot for each condition and plotted mean ± SEM. Statistical significance was assessed by ANOVA with Tukey's test. p-value < 0.05 was considered statistically significant. These data support the analysis performed in Figure 2.

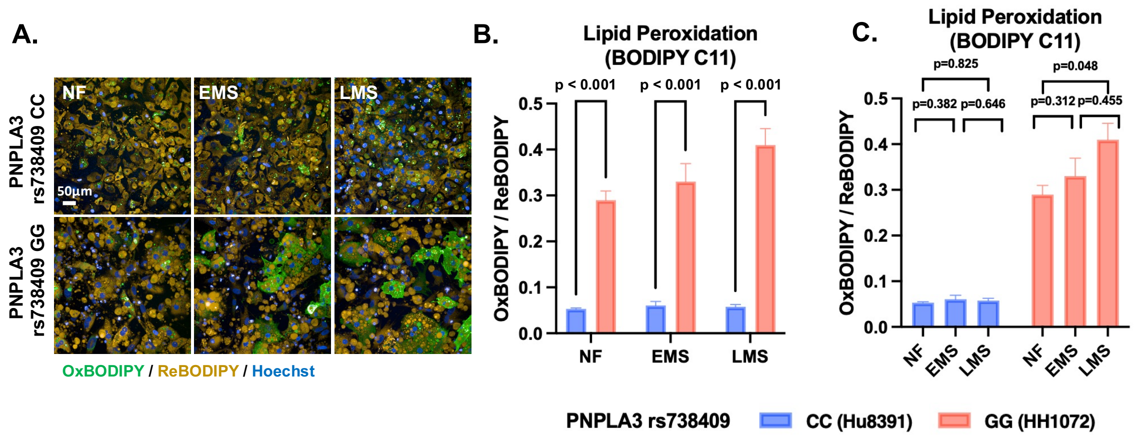

Fig S6. Increased lipid peroxidation (LPO) was observed in PNPLA3 rs738409 GG variant 96-well plate LAMPS compared to PNPLA3 CC wild type demonstrating the role of oxidative stress in genotype-specific MASLD progression. 96-well plate LAMPS were constructed using PNPLA3-genotyped hepatocytes and NPCs in NF, EMS and LMS media (9). Cells were labeled with BODIPY 581/591 C11 to monitor LPO in PNPLA3 GG variant and CC wild type models. The ratio of oxidized BODIPY (green) to reduced BODIPY (yellow) reflects the overall LPO level in each model. (A) Representative images of BODIPY 581/591 C11 staining in each PNPLA3 genotype and media condition, 40X; scale 50µm. (B) A significant increase in the ratio of OxBODIPY / ReBODIPY was observed in PNPLA3 GG variant 96-well plate LAMPS compared to the CC wild type in each media condition. (C) A significant increase in the ratio of OxBODIPY / ReBODIPY was observed in LMS medium compared to NF medium only in PNPLA3 GG 96-well plate LAMPS. Data were obtained on Day 5 with n = 4 wells from a single patient lot for each PNPLA3 genotype and plotted mean ± SEM for each media condition. Statistical significance was assessed by ANOVA with Tukey's test. p-value < 0.05 was considered statistically significant.

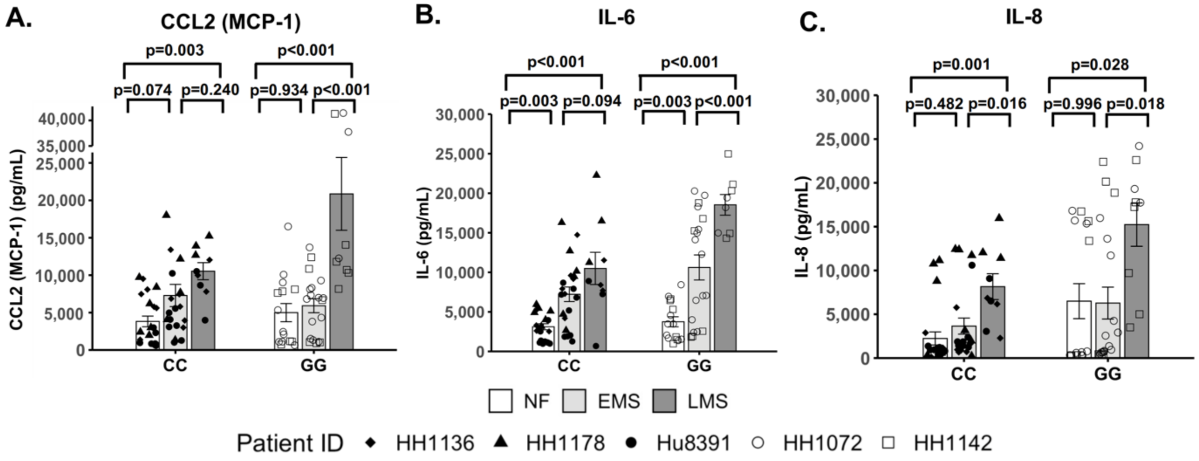

Figure S7. Increased pro-inflammatory cytokine secretion was observed in both EMS and LMS media compared to NF medium in PNPLA3 CC and GG LAMPS demonstrating lifestyle driven inflammation. (A-C) Significant increases in the secretion of CCL2 (A), IL-6 (B) and IL-8 (C) were observed in both EMS and LMS media compared to NF medium for each PNPLA3 genotype. While all three cytokines showed significant increases in LMS medium compared to EMS medium in PNPLA3 GG variant LAMPS (A-C), only IL-8 secretion was significantly increased in PNPLA3 CC wild type LAMPS (C) in LMS medium, consistent with recent studies demonstrating increased immune activation and inflammation associated with the PNPLA3 GG variant (10-12). Data were obtained on Day 8 with a minimum of n = 3 LAMPS from each patient lot for each condition and plotted mean ± SEM. Statistical significance was assessed by ANOVA with Tukey's test. p-value < 0.05 was considered statistically significant. These data support the analysis performed in Figure 3.

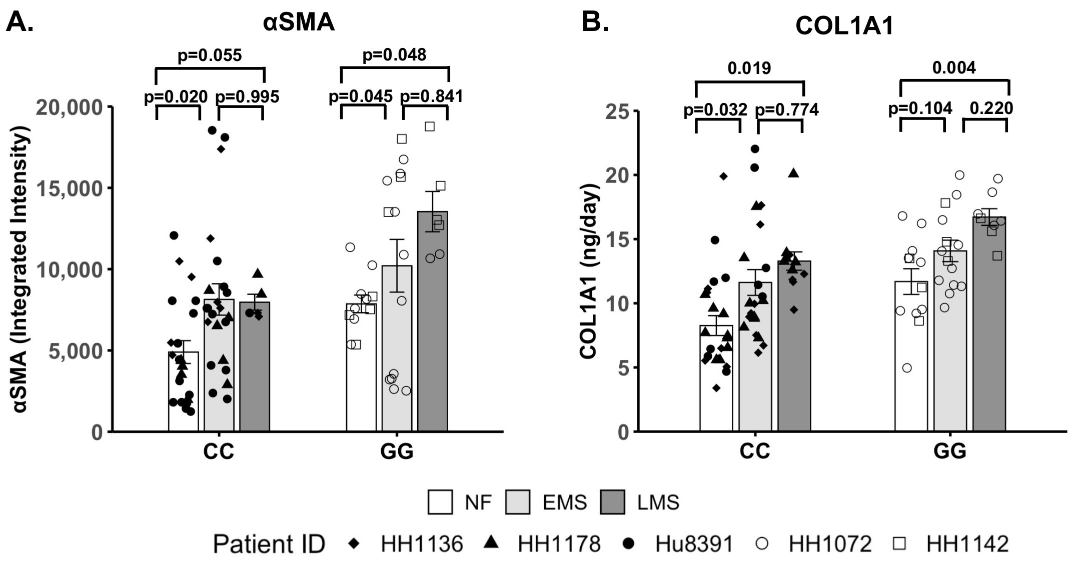

Figure S8. Increased stellate cell activation and COL1A1 secretion are observed in both EMS and LMS media compared to NF medium in PNPLA3 CC and GG LAMPS demonstrating the impact of lifestyle in addition to the genotype. (A) A significant increase in αSMA integrated intensity was observed between EMS and LMS compared to NF within each PNPLA3 genotype; however, no significant differences were observed between EMS and LMS media for either PNPLA3 genotype. (B) A significant increase in COL1A1 secretion was observed in LMS medium compared to NF medium for PNPLA3 genotypes. Data were obtained on Day 8 with a minimum of n = 3 LAMPS from each patient lot for each media condition and plotted mean ± SEM. Statistical significance was assessed by ANOVA with Tukey's test. p-value < 0.05 was considered statistically significant. These data support the analysis performed in Figure 4.

Table S3. Drug binding for resmetirom used in LAMPS studies.

**Media source**	**Resmetirom Concentration (μM)**
Media Blank	Not detected
Input media (t = 0 hr)	1.15 μM (0.500 μg/mL)
Chip #1 efflux (t = 72 hr)	1.25 μM (0.545 μg/mL)
Chip #2 efflux (t = 72 hr)	1.17 μM (0.511 μg/mL)

To assess the drug binding capability of the polydimethylsiloxane (PDMS)-containing LAMPS device for resmetirom (FW = 435.22), we used perfusion flow tests and mass spectrometry analysis of flow through collected from LAMPS devices at 72 h to determine the overall effective concentration of resmetirom compared to the starting concentration (input) of drug as previously described (3, 5, 9).

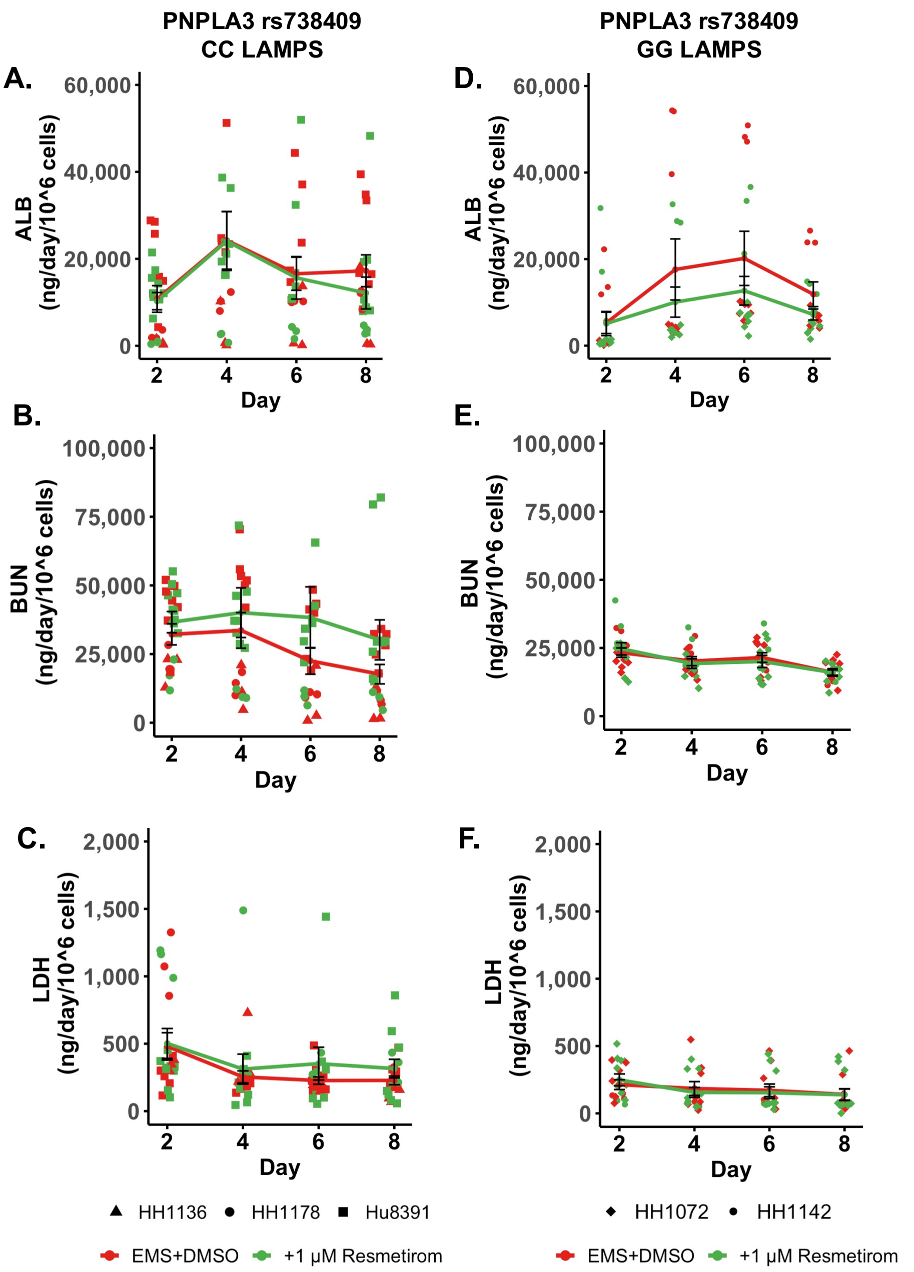

Figure S9. Similar model functionality and cytotoxicity profiles were observed in PNPLA3-LAMPS treated with resmetirom compared to vehicle control, demonstrating that there were no significant adverse effects of resmetriom treatment. For PNPLA3 CC wild type LAMPS (A-C) and PNPLA3 GG variant LAMPS (D-F), albumin (ALB; A and D), blood urea nitrogen (BUN; B and E) and lactate dehydrogenase (LDH; (C and F) secretion profiles were monitored to assess LAMPS functionality and cytotoxicity of 1 µM resmetirom treatment. No significant changes were observed between resmetirom treatment and vehicle control for ALB, BUN, or LDH secretion, demonstrating that drug treatment does not affect model functionality and cytotoxicity. Data were plotted mean ± SEM with a minimum of n = 3 LAMPS from each patient lot for each treatment condition. Statistical significance was assessed by ANOVA with Tukey's test for each indicated time point. p-values < 0.05 were considered statistically significant.

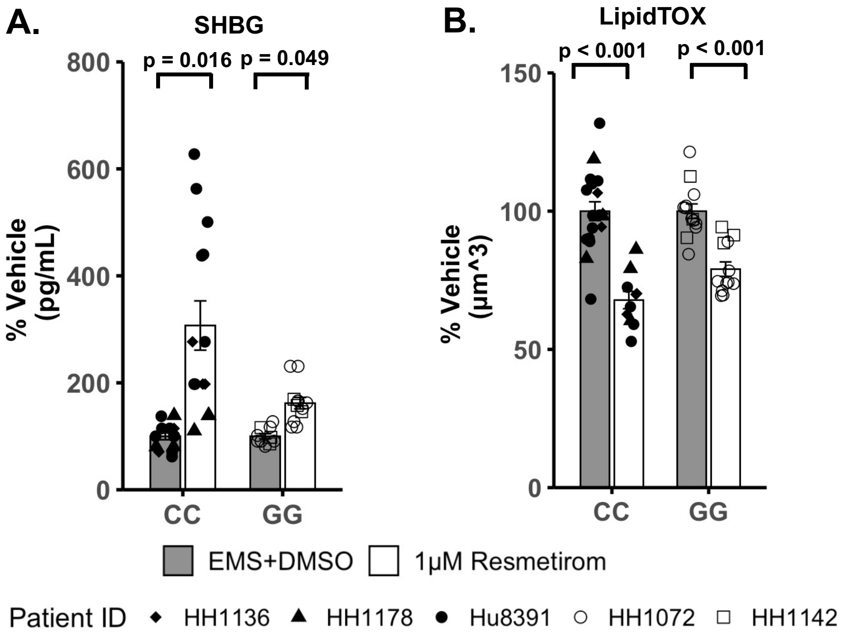

Figure S10. Resmetirom treatment resulted in increased SHBG secretion and steatosis reduction in both PNPLA3 GG variant and CC wild type LAMPS demonstrating the pharmacodynamic effect of resmetirom treatment. (A) Significantly increased SHBG secretion was observed in both PNPLA3 CC wild type and GG variant LAMPS with 1 µM resmetirom treatment. (B) A significant reduction in steatosis was observed in both PNPLA3 CC wild type and GG variant LAMPS with 1 µM resmetirom treatment compared to vehicle control. Data were obtained on Day 8 with a minimum of n = 3 LAMPS from each patient lot for each condition and plotted mean ± SEM. Statistical significance was assessed by ANOVA with Tukey's test. p-value < 0.05 was considered statistically significant. These data support the analysis performed in Figure 5 and are both consistent with recent clinical evidence demonstrating the pharmacodynamic effects of resmetirom (13, 14).

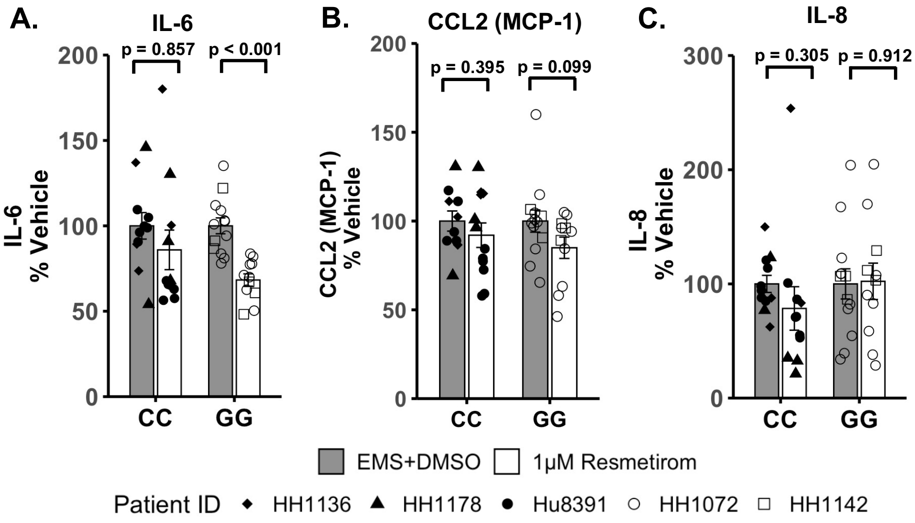

Figure S11. Resmetirom treatment significantly reduced the secretion of the pro-inflammatory cytokine IL-6 in PNPLA3 GG variant LAMPS. While no genotype-specific differences were observed in the reduction of secreted cytokines when the normalized values (normalized to NF medium) of PNPLA3 GG variant and CC wild type LAMPS were compared (Fig 3D-F), resmetirom treatment did result in a significant reduction of IL-6 in PNPLA3 GG variant LAMPS, but not in PNPLA3 CC wild type LAMPS when compared to their respective vehicle control (A). No significant reduction was observed for the secretion of CCL2 (B) or IL-8 (C) in either PNPLA3 LAMPS genotype when compared to their respective vehicle control. Data (% of vehicle control) were obtained on Day 8 with a minimum of n = 3 LAMPS from each patient lot for each condition and plotted ± SEM. Statistical significance was assessed by ANOVA with Tukey's test. p-values <0.05 were considered statistically significant.

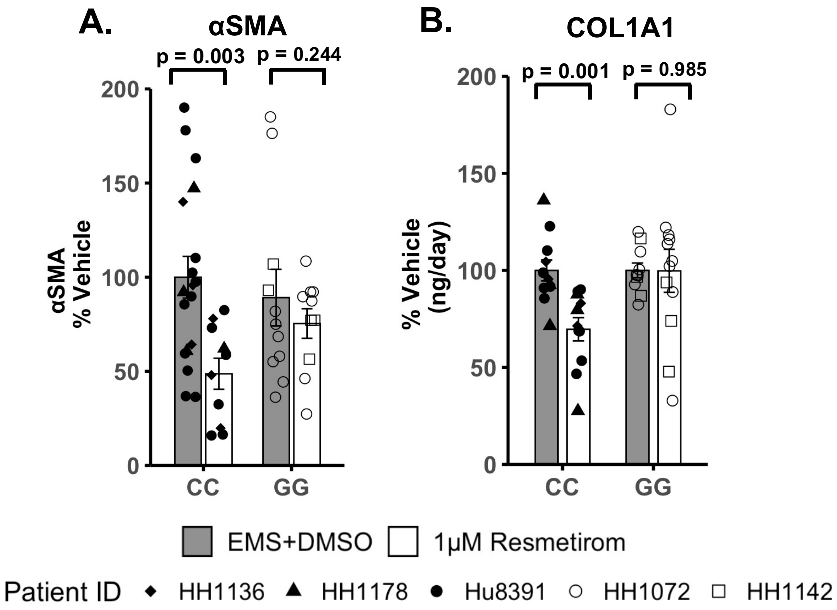

Figure S12. Resmetirom treatment resulted in a significant reduction in stellate cell activation and COL1A1 secretion in PNPLA3 CC wild type LAMPS but not in GG variant LAMPS demonstrating a genotype-specific drug response. (A and B) Compared to vehicle control, 1µM resmetirom treatment significantly reduced both αSMA integrated intensity (A) and the secretion of COL1A1 (B) in PNPLA3 CC wild type LAMPS, but not in GG variant LAMPS. Data were obtained on Day 8 with a minimum of n = 3 LAMPS from each patient lot for each condition and plotted mean ± SEM. Statistical significance was assessed by ANOVA with Tukey's test. p-value < 0.05 was considered statistically significant. These data support the analysis performed in Figure 7.

Supplemental Methods

LAMPS model assembly and maintenance workflow.

Day -3:

(a) *Mixed matrix coating of MPS devices:* The interior of the devices was dried under vacuum prior to protein coating with 100 µg/mL bovine fibronectin (Sigma Millipore) and 150 µg/mL rat-tail collagen, type 1(Corning), in PBS for 1 h at room temperature. The collagen/fibronectin solution was then removed, and devices were filled with PBS and stored at 4°C until use.
(b) *Differentiation of THP-1 cells:* THP-1 cells were treated with 200 ηg/mL phorbol myristate acetate (PMA; Sigma Millipore) to facilitate their differentiation into mature macrophages for seeding into LAMPS models on Day -1 (48 h treatment).

Day -2:

(a) *Hepatocyte seeding:* Cryopreserved hepatocytes were thawed following the manufacturer's recommendations. Hepatocytes were pelleted at 100 x g for 10 minutes using Cryopreserved Hepatocyte Recovery Medium (CHRM; ThermoFisher), and then resuspended at 2.75 x 10^6 hepatocytes/mL in hepatocyte plating media (HPM). Hepatocyte cell solution was then injected into the interstitial compartment of the device for overnight incubation at 37°C to allow for cell adherence and spreading.

Day -1:

(a) *LECM coating of MPS devices:* HPM was removed from the device and a solution of 400 µg/ml of porcine liver extracellular matrix prepared in NF media (LECM; a kind gift from Dr. Stephen Badylak's laboratory at the McGowan Institute for Regenerative Medicine, University of Pittsburgh) was added and incubated for 3 h at 37°C to create a thin matrix layer on top of the hepatocytes to mimic the Space of Disse.

(b) *LSEC and THP-1 seeding:* During the LECM incubation, LSEC and THP-1 cell suspensions are prepared in NF media for seeding into LAMPS. LSECs were thawed and a cell suspension was prepared at a concentration of 3.0 x 10^6 cells/mL. Differentiated THP-1 cells were prepared at a concentration of 1.6 x 10^6 cells/mL. The individual cell solutions were combined at a 1:1 ratio to yield final cell concentrations of 1.5 x 10^6 (LSEC) and 0.8 x 10^6 (THP-1) cells/mL. LECM solution was removed by gentle aspiration using a 1 mL syringe with a blunt needle (Fisher Scientific) and the LSEC/THP-1 cell solution was injected into each device and incubated for 2 h at 37°C.

(c) *Collagen/LX-2 overlay:* LX-2 cells were prepared at a concentration of 0.2 x 10^6 cells/mL and were suspended in 1 mL of a 2.5 mg/mL solution of pH 7.2 collagen I/10 mM HEPES/HBSS and injected into devices. The devices were then inverted for 1 h at 37°C during collagen polymerization to ensure an initial spatial separation of hepatocytes and LX-2 stellate cells.

The devices were then re-inverted and incubated overnight at 37°C. The collagen overlay functions to maintain hepatocyte morphology and functionality over extended culture time.

Day 0:

(a) *Establishment of flow:* The next day, flow was initiated using pressure driven pumps (KD Scientific) to perfuse media in glass syringes (Hamilton) at a flow rate of 5 (3-6% O_2) µL/hour to achieve a target oxygen concentration of 3-6% O_2, corresponding to zone 3 (hepatic venule) oxygen levels, as previously described (2). Devices were then maintained for 10 days at this flow rate.

(b) *Drug testing in LAMPS.* For drug studies, EMS media was prepared as described above and supplemented with the indicated concentration of drug (<0.1% DMSO v/v final concentration). EMS media containing drug was added at Day 0 during the initiation of flow for the duration of the experimental time course.

Supplemental References

1. Gough A, Soto-Gutierrez A, Vernetti L, Ebrahimkhani MR, Stern AM, Taylor DL. Human biomimetic liver microphysiology systems in drug development and precision medicine. Nature reviews Gastroenterology & hepatology. 2021;18(4):252-68.
2. Lee-Montiel FT, George SM, Gough AH, Sharma AD, Wu J, DeBiasio R, et al. Control of oxygen tension recapitulates zone-specific functions in human liver microphysiology systems. Exp Biol Med (Maywood). 2017;242(16):1617-32.
3. Lefever DE, Miedel MT, Pei F, DiStefano JK, Debiasio R, Shun TY, et al. A Quantitative Systems Pharmacology Platform Reveals NAFLD Pathophysiological States and Targeting Strategies. Metabolites. 2022;12(6).
4. Saydmohammed M, Jha A, Mahajan V, Gavlock D, Shun TY, DeBiasio R, et al. Quantifying the progression of non-alcoholic fatty liver disease in human biomimetic liver microphysiology systems with fluorescent protein biosensors. Exp Biol Med (Maywood). 2021;246(22):2420-41.
5. Vernetti LA, Senutovitch N, Boltz R, DeBiasio R, Shun TY, Gough A, Taylor DL. A human liver microphysiology platform for investigating physiology, drug safety, and disease models. Exp Biol Med (Maywood). 2016;241(1):101-14.
6. Chitturi S, Abeygunasekera S, Farrell GC, Holmes-Walker J, Hui JM, Fung C, et al. NASH and insulin resistance: Insulin hypersecretion and specific association with the insulin resistance syndrome. Hepatology. 2002;35(2):373-9.
7. Kim NH, Kim DL, Choi KM, Baik SH, Choi DS. Serum insulin, proinsulin and proinsulin/insulin ratio in type 2 diabetic patients: as an index of beta-cell function or insulin resistance. Korean J Intern Med. 2000;15(3):195-201.
8. Sanyal AJ, Campbell-Sargent C, Mirshahi F, Rizzo WB, Contos MJ, Sterling RK, et al. Nonalcoholic steatohepatitis: association of insulin resistance and mitochondrial abnormalities. Gastroenterology. 2001;120(5):1183-92.
9. Miedel MT, Gavlock DC, Jia S, Gough A, Taylor DL, Stern AM. Modeling the Effect of the Metastatic Microenvironment on Phenotypes Conferred by Estrogen Receptor Mutations Using a Human Liver Microphysiological System. Sci Rep. 2019;9(1):8341.
10. Kabbani M, Michailidis E, Steensels S, Fulmer CG, Luna JM, Le Pen J, et al. Human hepatocyte PNPLA3-148M exacerbates rapid non-alcoholic fatty liver disease development in chimeric mice. Cell Rep. 2022;40(11):111321.
11. Kostrzewski T, Snow S, Battle AL, Peel S, Ahmad Z, Basak J, et al. Modelling human liver fibrosis in the context of non-alcoholic steatohepatitis using a microphysiological system. Commun Biol. 2021;4(1):1080.
12. Krawczyk M, Liebe R, Lammert F. Toward Genetic Prediction of Nonalcoholic Fatty Liver Disease Trajectories: PNPLA3 and Beyond. Gastroenterology. 2020;158(7):1865-80 e1.
13. Harrison SA, Bedossa P, Guy CD, Schattenberg JM, Loomba R, Taub R, et al. A Phase 3, Randomized, Controlled Trial of Resmetirom in NASH with Liver Fibrosis. N Engl J Med. 2024;390(6):497-509.
14. Harrison SA, Taub R, Neff GW, Lucas KJ, Labriola D, Moussa SE, et al. Resmetirom for nonalcoholic fatty liver disease: a randomized, double-blind, placebo-controlled phase 3 trial. Nat Med. 2023;29(11):2919-28.
